# ALS-associated mutant FUS induces selective motor neuron degeneration through toxic gain of function

**DOI:** 10.1038/ncomms10465

**Published:** 2016-02-04

**Authors:** Aarti Sharma, Alexander K. Lyashchenko, Lei Lu, Sara Ebrahimi Nasrabady, Margot Elmaleh, Monica Mendelsohn, Adriana Nemes, Juan Carlos Tapia, George Z. Mentis, Neil A. Shneider

**Affiliations:** 1 Department of Neurology, Center for Motor Neuron Biology and Disease, Columbia University, 630 W 168th Street, P&S Building, Room 5-423, New York, New York 10032, USA; 2 Department of Pathology and Cell Biology, Center for Motor Neuron Biology and Disease, Columbia University, New York, New York 10032, USA; 3 Department of Neuroscience, Howard Hughes Medical Institute, Columbia University, New York, New York 10032, USA; 4 Department of Neuroscience, Columbia University, New York, New York 10032, USA

## Abstract

Mutations in FUS cause amyotrophic lateral sclerosis (ALS), including some of the most aggressive, juvenile-onset forms of the disease. FUS loss-of-function and toxic gain-of-function mechanisms have been proposed to explain how mutant FUS leads to motor neuron degeneration, but neither has been firmly established in the pathogenesis of ALS. Here we characterize a series of transgenic FUS mouse lines that manifest progressive, mutant-dependent motor neuron degeneration preceded by early, structural and functional abnormalities at the neuromuscular junction. A novel, conditional FUS knockout mutant reveals that postnatal elimination of FUS has no effect on motor neuron survival or function. Moreover, endogenous FUS does not contribute to the onset of the ALS phenotype induced by mutant FUS. These findings demonstrate that FUS-dependent motor degeneration is not due to loss of FUS function, but to the gain of toxic properties conferred by ALS mutations.

## Abstract (web summary)

The mechanism by which FUS mutations cause familial ALS remains unclear. Here, the authors use mouse transgenic models to show that a toxic gain-of-function underlies motor neuron degeneration, and that the toxicity of mutant FUS does not depend on a loss or excess of FUS activity.

## Graphical abstract

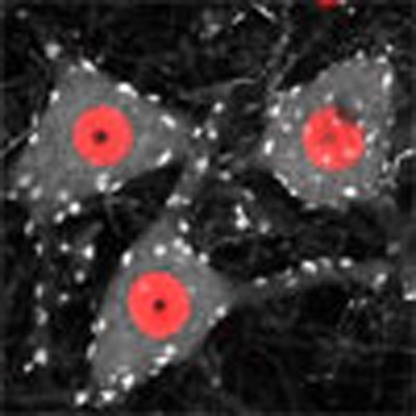

Amyotrophic lateral sclerosis (ALS) is a fatal neurological disorder characterized primarily by the rapid degeneration of motor neurons (MNs). Mutations in over 20 genes account for about 10% of cases^1^, but how mutations in each of these functionally diverse genes cause selective MN degeneration is unknown. The identification of the DNA/RNA-binding protein TDP-43 as a component of abnormal, cytoplasmic inclusions in sporadic ALS patients^2^ and the later discovery of ALS-causing mutations in the TDP-43 gene (*TARDBP*)^3^ led to the identification of causal mutations in the gene encoding the related protein *FU*sed in *S*arcoma (FUS)^4^,^5^. Together, these findings suggested that aberrant RNA processing may underlie common mechanisms of neurodegeneration in ALS^6^ and focused attention on the normal activities of TDP-43, FUS and other ALS-associated RNA-binding proteins^7^.

Over 50 mutations in *FUS* have been found in ALS families^8^, the majority of which are dominant, missense changes clustered in and around the C-terminal nuclear localization signal^9^. *FUS* mutations are associated with a broad range of clinical phenotypes including some of the most aggressive, juvenile-onset forms of the disease^10^. How mutant FUS causes ALS is unknown, but both gain- and loss-of-function mechanisms have been proposed^8^. The toxic gain of function may relate to the formation of abnormal aggregates of FUS in the nucleus and cytoplasm of affected neurons and glia in ALS patients with FUS mutations^11^,^12^. Alternatively, an excess of FUS activity may lead to MN degeneration, as suggested by the identification of non-coding mutations that increase the levels of non-mutant FUS^13^ in ALS patients and by the finding that overexpression of wild-type (WT) FUS in mice causes MN degeneration^14^.

FUS is a predominantly nuclear protein that plays multiple roles in DNA damage repair and in RNA transcription, splicing, transport and translation^15^,^16^. In neurons, FUS is also localized to dendrites and accumulates at excitatory synapses as an RNA–protein complex^17^ associated with N-methyl-D-aspartate receptor (NMDA) receptors^18^ and in RNA transporting granules in the soma and dendrites^19^. These data suggest that FUS, like TDP-43 (ref. 20), could play a role in the modulation of synaptic activity in the central nervous system by regulating mRNA transport and local translation in neurons. Whether any of these diverse activities of FUS is required for MN survival is not known. Thus, the role of FUS function in ALS remains to be determined.

To study the mechanisms of mutant FUS-mediated MN degeneration, we generated an allelic series of targeted, conditional transgenic mice in which a single-copy, WT or ALS-associated mutant human FUS (hFUS) is conditionally expressed from the *MAPT* (tau) locus. Analysis of these mutants reveals progressive, age- and mutation-dependent MN degeneration that faithfully models several key aspects of the ALS phenotype, including selectivity for MN subtypes most vulnerable in the human disease. MN loss in this model is associated with early synaptic failure and withdrawal of the motor axon from the neuromuscular junction. We demonstrate that expression of mutant hFUS is sufficient to cause MN degeneration, and that alleles associated with aggressive, juvenile-onset forms of ALS are more pathogenic in our disease models.

To determine whether the toxicity of mutant hFUS is a consequence of FUS loss of function, we generated a conditional FUS knockout mouse to demonstrate that long-term survival of MNs *in vivo* is not dependent on postnatal FUS. These data provide genetic evidence that MN degeneration in ALS-FUS is not a consequence of FUS loss of function, but a toxic gain of function conferred by ALS-associated FUS mutations. To test whether this novel toxic function depends on the presence of WT FUS, we combined the FUS transgenic and knockout mutants to express exogenous mutant FUS in the context of reduced postnatal FUS. These studies demonstrate *in vivo* that the ALS mutant hFUS is not dependent on endogenous FUS to initiate MN degeneration, arguing against a ‘seeding' mechanism^21^ in which mutant hFUS interacts with WT FUS to induce the formation of toxic aggregates. Moreover, the finding that FUS elimination has no effect on the onset of MN degeneration in our model argues that an excess of FUS activity alone does not cause MN degeneration. Together, these data support a disease model in which ALS mutant *FUS* causes MN degeneration through a toxic gain of function mechanism that does not involve the normal activity of FUS.

## Results

### Generation and characterization of ALS-FUS mutant mice

**Figure 1:**
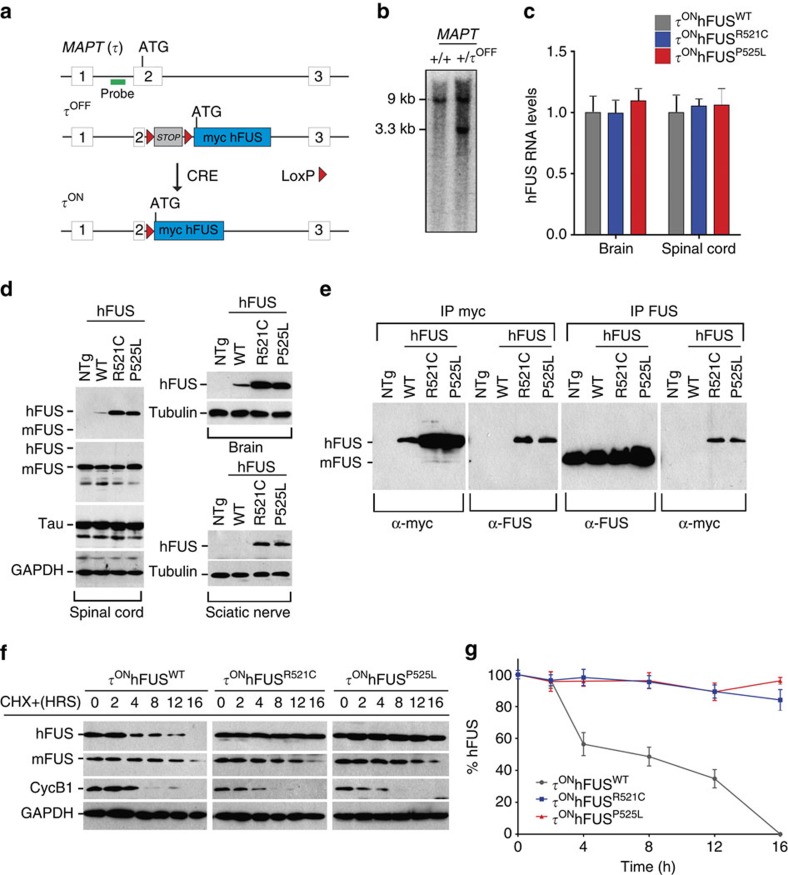
Conditional expression of myc-tagged hFUS from the *MAPT* locus reveals increased stability of ALS mutant protein. (**a**) Organization of the endogenous (τ) and targeted (τ^OFF^) *MAPT* locus on mouse Chromosome 11. An hFUS expression cassette (light blue) was introduced into exon 2 of *MAPT*, replacing the endogenous start codon. Excision of the LOX-STOP-LOX transcriptional stop sequence by Cre-mediated recombination allows expression of the myc-tagged hFUS^WT^, hFUS^R521C^ and hFUS^P525L^ cDNAs from the τ^ON^ allele of the *MAPT* locus. (**b**) Southern blot analysis of targeted ES cell clones following *Bam*HI digestion and hybridization with a 5′ external probe (green, **a**). Wild-type ES cells (left, +/+) display a 9.0-kb band. Targeted ES cells (right, +/τ^OFF^) yielded a 3.3-kb band corresponding to the τ^OFF^ allele in addition to the 9.0-kb band corresponding to the untargeted allele at the *MAPT* locus. (**c**) RT–qPCR analysis of hFUS transcripts in total brain and total spinal cord (p90, *N*=4). Data are represented as mean and s.e.m. (**d**) Western blot analysis of total spinal cord, brain and sciatic nerve extracts. 7x myc-tagged hFUS migrated at a higher molecular weight (90 kDa) relative to endogenous mouse FUS (73 kDa). Immunoblotting with anti-FUS antibody did not detect myc-hFUS. All mutants analysed were heterozygous for the τ^ON^hFUS alleles (with the exception of Supplementary Fig. 4A–C) and tau expression was maintained in these animals. (**e**) Immunoprecipitation of P90 mouse spinal cord extracts with anti-myc or anti-FUS antibodies. myc-hFUS bands were detected with an anti-FUS antibody following immunoprecipitation with an anti-myc antibody. (**f**) Western blot analysis of cyclohexamide (CHX)-treated embryonic stem cell-derived motor neurons (ESMNs). At 16 h post CHX treatment, myc-hFUS was no longer detected with anti-myc antibody in cells derived from τ^ON^hFUS^WT^ animals but persisted in τ^ON^hFUS^R521C^ and τ^ON^hFUS^P525L^ samples. Levels of endogenous mouse FUS detected with anti-FUS antibody were similarly decreased in all samples. CyclinB1 was used as an internal positive control for CHX treatment efficacy. (**g**) Quantification of myc-hFUS levels, normalized to myc-hFUS levels at 0 h, in CHX-treated ESMNs. Data are represented as mean and s.e.m. of *N*=3 differentiations.

To investigate the mechanisms by which ALS-associated mutations in FUS cause MN degeneration, we generated a series of targeted transgenic mouse lines expressing WT or ALS-associated, mutant human FUS from the mouse *MAPT* (tau (τ)) locus^22^. cDNAs encoding WT human FUS (hFUS^WT^) or one of two mutant isoforms—hFUS^R521C^ associated with typical, adult-onset ALS, and hFUS^P525L^ found in patients with an aggressive, juvenile-onset form of the disease^10^—were introduced (Fig. 1a,b) into the first coding exon of tau, preceded by an N-terminal myc tag to label the exogenous hFUS protein. The τ*-hFUS* alleles were made conditional by placing a transcriptional stop flanked by loxP sites 5′ of the hFUS cDNA (Fig. 1a) so that the recombinant *tau* allele is silent until activated by *Cre*-mediated recombination (Supplementary Fig. 1A). The three conditional τ-myc-hFUS mouse lines are referred to as τ^OFF^hFUS^WT^, τ^OFF^hFUS^R521C^ and τ^OFF^hFUS^P525L^.

To express hFUS constitutively in all τ-expressing cells, we crossed the three τ^OFF^ conditional mouse lines to the *Prm1-Cre*^23^ germline CRE deleter to generate the τ^ON^hFUS^WT^, τ^ON^hFUS^R521C^ and τ^ON^hFUS^P525L^ lines. In each of the three, heterozygous τ^ON^hFUS mouse lines, no significant change in the level of endogenous mouse FUS (mFUS) or tau was observed (Fig. 1d). Compared with endogenous mouse FUS (mFUS), the slowly migrating (90 kDa) myc-hFUS protein was expressed at low levels and could not be detected by western blot analysis of spinal cord extracts by any one of a series of FUS antibodies (Fig. 1d; antibodies listed in Supplementary Table 1). However, after enrichment by myc or FUS immunoprecipitation followed by western blot analysis with a FUS or myc antibody, respectively, exogenous hFUS was detected in the τ^ON^hFUS^R521C^ and τ^ON^hFUS^P525L^ samples (Fig. 1e).

Western blot analysis also showed that the levels of hFUS^R521C^ and hFUS^P525L^ were ∼4 times higher than hFUS^WT^ in the spinal cord and brain (Fig. 1d). All hFUS transcript levels were equivalent in brain and spinal cord of the three lines (Fig. 1c; primers listed in Supplementary Table 2), so this increase could not be explained at the level of transcription. However, cyclohexamide blockade of translation in embryonic stem cell-derived MNs from each of the three τhFUS mutants revealed decreased turnover of the mutant protein (Fig. 1f,g). Together these data demonstrate that the accumulation of mutant versus WT hFUS is due to increased stability of the ALS-associated mutant proteins.

### Mislocalization of ALS mutant human FUS in MNs

**Figure 2:**
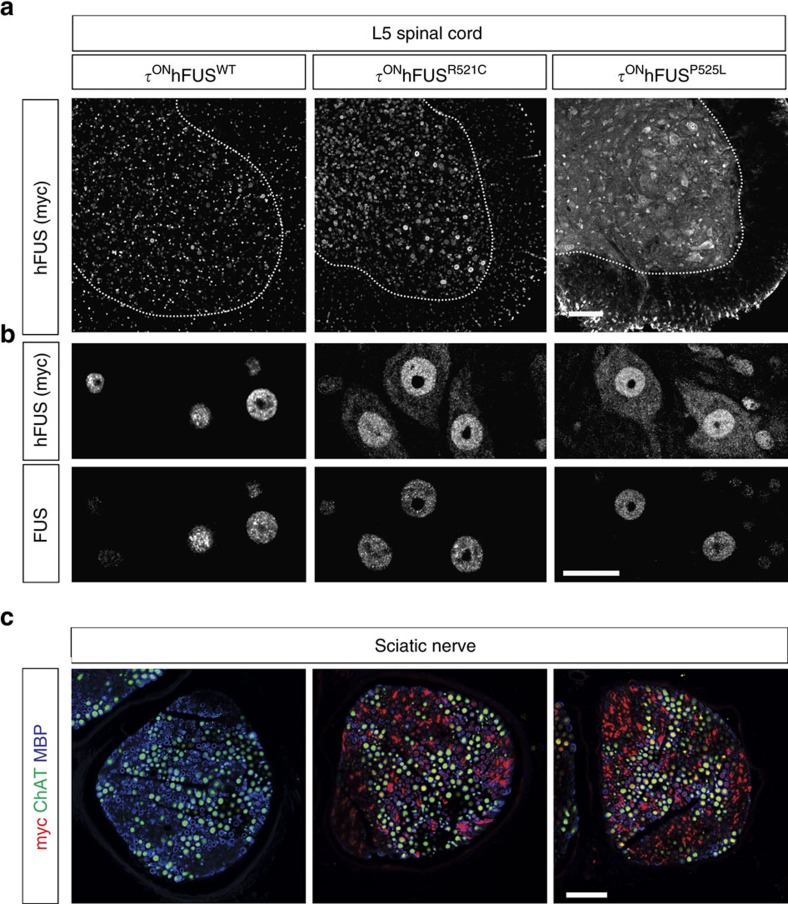
Diffuse cytoplasmic mislocalization of mutant FUS extends to sensory and motor axons in the peripheral nerve. (**a**) Confocal images of L5 spinal cord cross-sections of hFUS^WT^ (left), ALS mutant R521C (centre) and P525L (right) animals immunostained with anti-myc and anti-FUS antibodies. hFUS was detected by anti-myc antibodies in both the grey and the white matter (boundary marked by the dotted white line), consistent with the normal pattern of Tau (*MAPT*) expression in all neurons and in subpopulations of glia, including oligodendroglia and astrocytes^25^,^26^,^27^. Scale bar, 100 μm. (**b**) Higher magnification confocal images of L5 MNs, as in **a**. hFUS-WT protein was detected with anti-myc antibody (top, left) exclusively in the nucleus, whereas hFUS-R521C (top, centre) and hFUS-P525L (top, right) were detected both in the nucleus and in the cytoplasm of the MNs. Immunohistochemical staining with anti-FUS antibody (bottom) detected nuclear but not cytoplasmic FUS in all samples. Scale bar, 25 μm. (**c**) Cross-sections of sciatic nerves stained with anti-myc (red), anti-ChAT (green) and anti-MBP (myelin basic protein, blue) antibodies. hFUS-R521C and hFUS-P525L proteins, but not hFUS-WT, were detected in motor and sensory axons in the peripheral sciatic nerve. Scale bar, 40 μm.

In ALS patients with mutations in *FUS*, the predominantly nuclear FUS protein is mislocalized to the cytoplasm of neurons and glia^24^. Immunohistochemical analysis of the τ^ON^hFUS mutants demonstrated expression of myc-hFUS in neurons and non-neuronal cells (Fig. 2a) in a pattern consistent with the known pattern of MAPT expression in the spinal cord^25^,^26^,^27^,^28^. In the myc-hFUS^WT^ control mice, both hFUS and mFUS remained predominantly nuclear (Fig. 2a,c), however, in the τ^ON^hFUS^R521C^ and τ^ON^hFUS^P525L^ mutants, myc-hFUS was found mislocalized to the cytoplasm and dendrites of τ-expressing MNs (Fig. 2a,b), and mutant hFUS protein was also detected peripherally in the sciatic nerve (Figs 1d and 2c). Notably, the more pathogenic hFUS^P525L^ protein mislocalized to a greater degree compared with hFUS^R521C^, as shown by a higher cytoplasmic/nuclear ratio (Supplementary Fig. 2B). In contrast, endogenous mFUS remained localized to the nucleus and below levels of detection in the cytoplasm of the τ^ON^hFUS^R521C^ and τ^ON^hFUS^P525L^ mutants. Despite the marked cytoplasmic mislocalization of mutant hFUS, we observed no large, discrete cytoplasmic aggregates in lumbar spinal MNs in the τ^ON^hFUS^R521C^ or hFUS^P525L^ mice. These data demonstrate that ALS-associated mutations in hFUS result in the marked mislocalization of FUS in the cytoplasm, axon and dendrites of neurons, modelling a pathological hallmark of ALS-FUS. Moreover, the degree of mislocalization of hFUS^P525L^ versus hFUS^R521C^ in this mouse model correlates with the relative toxicity of the respective mutant FUS alleles in ALS patients.

### Selective MN loss and weakness in ALS-FUS mutants

**Figure 3:**
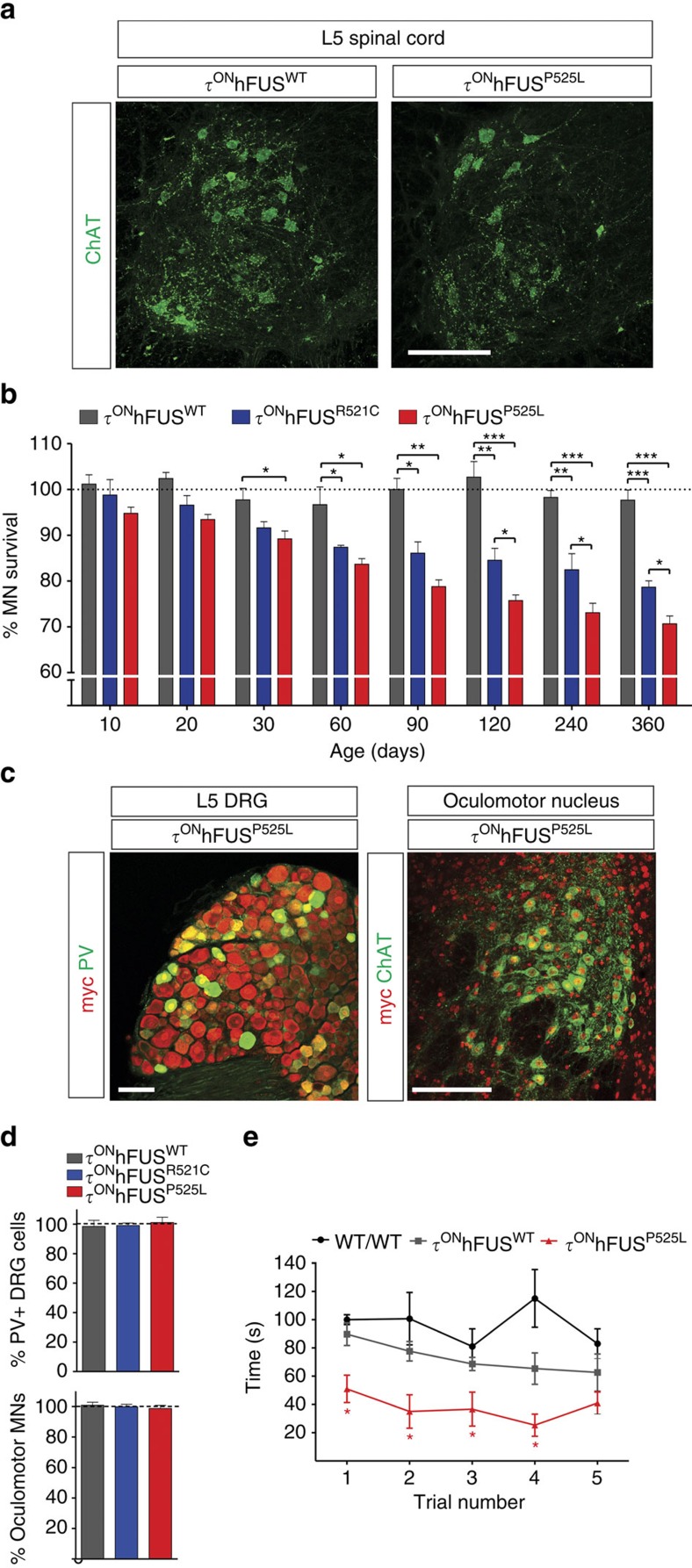
Progressive and selective loss of motor neurons in the lumbar spinal cord of mice expressing ALS mutant FUS. (**a**) Representative images of L5 spinal cord cross-sections of control τ^ON^hFUS^WT^ and τ^ON^hFUS^P525L^ p360 animals immunostained with anti-ChAT antibody. Scale bar, 100 μm. (**b**) MN survival in τ^ON^hFUS^WT^ (grey), τ^ON^hFUS^R521C^ (blue) and τ^ON^hFUS^P525L^ (red) animals calculated from the number of MNs in the L5 spinal cord normalized to that of non-transgenic wild-type controls (expressed as a percentage). (**c**) Representative images of parvalbumin-positive sensory neurons (PV, green, left) in the L5 DRG and MNs (ChAT, green, right) in the oculomotor nucleus in τ^ON^hFUS^P525L^ animals. Scale bar, 50 μm (left), 100 μm (right). (**d**) The number of parvalbumin-positive sensory neurons in the L5 DRG (left) and the number of MNs in the oculomotor nucleus (right) in p360 τ^ON^ animals normalized to non-transgenic wild-type controls (expressed as a percentage). (**e**) Evaluation of hind-limb motor function at p360 using the wire hang test. The latency for animals to fall was recorded over five trials with a 2-min rest period between each trial. (For **b**, **d** and **e**: *N*=4, *P<0.05, **P<0.01 and ***P<0.001 using one-way analysis of variance with Bonferroni's *post hoc* test. Error bars represent s.e.m.).

To determine if ALS mutant FUS expression in the τ^ON^hFUS^R521C^ and hFUS^P525L^ mice was associated with MN degeneration, we used an antibody against choline acetyl transferase (ChAT) to visualize and count spinal MNs at lumbar level 5 (L5; Fig. 3a). At postnatal day 10 (p10), there was no significant difference in the number of ChAT+ L5 MNs in the mutants compared with age-matched τ^ON^hFUS^WT^ and WT littermate controls (Fig. 3b), demonstrating that expression of hFUS^R521C^ or hFUS^P525L^ had no developmental effect on the number of MNs generated. We observed the first significant loss of MNs at p30 in the τ^ON^hFUS^P525L^ mutant, but no significant MN loss in the τ^ON^hFUS^R521C^ at that age (Fig. 3b). However, the number of ChAT+ MNs was significantly reduced in both the τ^ON^hFUS^R521C^ and τ^ON^hFUS^P525L^ mice at p60 (Fig. 3b). MN loss progressed steadily in both mutants to p360, but was more pronounced in the τ^ON^hFUS^P525L^ mutant (∼30%), compared with the τ^ON^hFUS^R521C^ mice (∼20%; Fig. 3b), demonstrating that the mutant allele associated with juvenile-onset ALS is also more pathogenic in this mouse model of the disease. Moreover, only mutant hFUS was toxic to MNs in this system as, in contrast, no significant loss of L5 MNs was observed in the τ^ON^hFUS^WT^ control (Fig. 3b).

To determine if neuronal loss was selective to MNs in these mutants, we counted parvalbumin (PV)-positive, proprioceptive sensory neurons in the L5 dorsal root ganglia and found no significant difference at p360 in the τ^ON^hFUS^P525L^ or FUS^R521C^ mutants relative to controls (Fig. 3c,d). Also we found no significant MN loss at p360 in oculomotor nucleus (Fig. 3c,d), a MN subpopulation spared in ALS patients and in the mutant SOD1 model of disease^29^. However, as in ALS patients and the SOD1^G93A^ mouse^30^,^31^,^32^,^33^,^34^, MN degeneration in the τ^ON^hFUS mutants was associated with marked astrocytosis and microgliosis, evidenced by an increase in both glial fibrillary acidic protein (GFAP) and Iba1 immunoreactivity in the ventral horn of the τ^ON^hFUS^R521C^ and hFUS^P525L^ mutants relative to the τ^ON^hFUS^WT^ controls (Supplementary Fig. 1B).

Expression of mutant hFUS had no effect on the survival of the τ^ON^hFUS mice; however, MN loss in the lumbar spinal cord was also associated with significant hind-limb weakness. Wire hang analysis at p360 demonstrated that the average latency to fall for the τ^ON^hFUS^R525L^ mutant was approximately half that of the τ^ON^hFUS^WT^ and WT controls (Fig. 3e).

These data demonstrate that ALS mutant hFUS, but not the WT protein, is toxic to MNs in this model system, and that hFUS^P525L^, the more pathogenic allele in patients, also causes earlier and more pronounced MN loss in the mouse compared with hFUS^R521C^. Moreover, neurodegeneration in the τ^ON^hFUS mutants is selective to MNs and associated with significant motor dysfunction, reproducing key aspects of the ALS phenotype.

### Early preferential denervation of fast twitch limb muscles

In ALS patients, fast-fatigable (FF) motor units are the earliest affected,^35^ and neurogenic changes in muscle can be observed before MN loss^36^. Similarly, MN loss in the SOD1 mouse is also preceded by denervation of skeletal muscles, with early and preferential involvement of FF motor units^37^,^38^. To determine whether expression of mutant hFUS results in the early withdrawal of motor axons from muscles innervated by FF motor units, we looked for evidence of denervation in three hind-limb muscles innervated by L5 MNs, the tibialis anterior (TA) innervated predominantly by FF MNs, the soleus (Sol) innervated mostly by slow (S) MNs and the gastrocnemius (GS) innervated by a mixture of S, fast fatigue resistant (FR) and FF motor units^39^.

**Figure 4:**
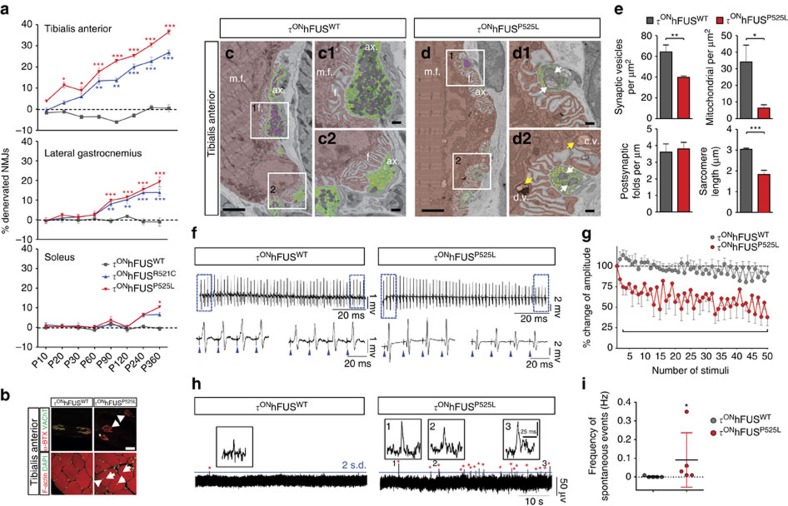
Mutant FUS causes early synaptic changes at the neuromuscular junction, leading to progressive and selective denervation. (**a**) Denervated NMJs in the TA, GS and SOL of τ^ON^hFUS^WT^ (grey), τ^ON^hFUS^R521C^ (blue) and τ^ON^hFUS^P525L^ (red) animals normalized to controls. *N*=4. *P<0.05, **P<0.01 and ***P<0.001 using one-way analysis of variance with Bonferroni's *post hoc* test. Error bars represent s.e.m. (**b**) Co-localization of VAChT-positive motor terminals (green) with acetylcholine receptors (α-BTX, red) as a marker of innervated endplates. In τ^ON^hFUS^P525L^ animals, denervated endplates (arrows, top right), partially innervated endplates (asterisk, top right) and internal nuclei (arrows bottom left) were observed. Scale bar, 40 μm (top), 20 μm (bottom). (**c**,**d**) Electron micrographs from TA NMJs in p30 τ^ON^hFUS^WT^ (**c**) and τ^ON^hFUS^P525L^ (**d**) animals, show that motor terminals (ax., green) were correctly opposed to postsynaptic folds (**f**) in the muscle (brown, m.f). Higher magnification of boxes 1 and 2 are shown in c_1_, d_1_ and c_2_, d_2_. In τ^ON^hFUS^P525L^ animals, the distributions of mitochondria (purple) and synaptic vesicles (yellow) were altered. Pre- (white arrows, d_1_, d_2_) and post-synaptic structurally damaged mitochondria (yellow arrows, d_2_) with clear- and dark-vacuoles (d.v. and c.v., d_2_) were observed in τ^ON^hFUS^P525L^animals. Scale bar, 1 μm (**c**,**d**), 0.4 μm (for c_1_, c_2_ and d_1_,d_2_). (**e**) Quantification of synaptic vesicles, healthy mitochondria, postsynaptic folds and sarcomere length in NMJs from τ^ON^hFUS^WT^ (grey) and τ^ON^hFUS^P525L^ animals (red). *N*=6 junctions, 2 animals. (**f**) EMG responses in the TA following a 50-Hz, 1 s stimulation of the sciatic nerve in τ^ON^hFUS^WT^ (left) and τ^ON^hFUS^P525L^ (right) animals. The first and last four responses are shown in an expanded time scale below. Blue triangles mark the stimulus artefact. (**g**) M-responses at subsequent stimuli, normalized to the first response in τ^ON^hFUS^WT^ (grey) and τ^ON^hFUS^P525L^ (red) animals. (*N*=5. *P<0.05 using two-way analysis of variance with Fisher's LSD *post-hoc* test. Error bars represent s.e.m.). (**h**) Spontaneous activity in the TA of τ^ON^hFUS^WT^ (left) and τ^ON^hFUS^P525L^ (right) animals. Examples of spontaneous activity are shown in insets 1–3. (**i**) Frequency of spontaneous events in τ^ON^hFUS^P525L^ (red) and τ^ON^hFUS^WT^ (grey) animals. (*N*=5. *P<0.01 using *t*-test. Error bars represent s.e.m.).

Using an antibody against vesicular acetyl choline transporter (VAChT) to visualize motor axon terminals and a fluorescent conjugate of α-bungarotoxin (α-BTX) to label the post-synaptic surface of the neuromuscular junction (NMJ), we found no significant denervation of NMJs at p10 in any of the three τ^ON^hFUS mice (Fig. 4a,b). By p20, however, before MN loss was detected at L5, a significant number of NMJs (11.4%) in the TA of the τ^ON^hFUS^P525L^ mutants had no associated input (Fig. 4a), demonstrating early retraction of motor axons. Similarly, in the τ^ON^hFUS^R521C^ mice, we observed significant denervation of the TA at p40 before L5 MN loss at p60 (Fig. 3b). Denervation of the GS followed at p90 when ∼10% of NMJs were vacant in both the τ^ON^hFUS^R521C^ and τ^ON^hFUS^P525L^ mice (Fig. 4a). Denervation in both the TA and GS progressed steadily in these mutants so that at p360 in the τ^ON^hFUS^R521C^ mouse, 30.2% and 13.9% of endplates were vacant in the TA and GS, respectively (Fig. 4a). Similarly, in the τ^ON^hFUS^P525L^ mice 36.7% and 19.3% of NMJs were denervated in the TA and GS, respectively (Fig. 4a). In the predominantly slow Sol muscle, no significant denervation was noted at 1 year (p360) in the τ^ON^hFUS^R521C^, however, over 10% of Sol motor terminals were vacant in the τ^ON^hFUS^P525L^ mutant at that time point (Fig. 4a). As further evidence of denervation, internal nuclei (Fig. 4b) and decreased fibre diameter (Supplementary Fig. 3B) were also observed in the TA of the τ^ON^hFUS^P525L^ mutant. The same results were found using other pre-synaptic markers including synaptophysin, neurofilament and synaptic vesicle protein 2, demonstrating that the apparent denervation was not a consequence of VAChT downregulation in the mutants (Supplementary Fig. 3A). As τ^ON^hFUS is not expressed in the muscle, these data show that mutant FUS causes the early retraction of motor axons from the neuromuscular junction by a pre-synaptic mechanism, and that this pathology preferentially involves the motor units known to be most vulnerable in ALS.

As was the case with MN soma counts, no denervation of the TA, GS or Sol muscles was observed in the τ^ON^hFUS^WT^ control, demonstrating that the phenotype was specific to mutant hFUS. Here we also used TA denervation as a sensitive measure of MN loss to test whether the selective toxicity of hFUS^P525L^ and hFUS^R521C^ is a result of higher steady-state levels of mutant versus WT hFUS. We analysed mutant mice that were homozygous for the τ^ON^hFUS^WT^ or τ^ON^hFUS^P525L^ allele. In each case, the level of hFUS RNA determined by reverse transcription–quantitative PCR (RT–qPCR) was double in the homozygous mutant compared with the respective heterozygous control (Supplementary Fig. 4A). Western blot analysis of the homozygous animals confirmed that the level of hFUS protein was twofold higher than in the corresponding heterozygote mutant, which was still too low to negatively regulate endogenous mFUS expression (Supplementary Fig. 4B). In the homozygous animals, the relative levels of hFUS^P525L^ and FUS^WT^ remained approximately 4 to 1 (Supplementary Fig. 4B). No denervation of the TA NMJs was detected in the homozygous τ^ON^hFUS^WT^ animals at p90 (Supplementary Fig. 4C), demonstrating that hFUS^WT^ even at twice the level expressed in the τ^ON^hFUS^WT^ heterozygotes is not toxic to MNs. Further, analysis of the τ^ON^hFUS^P525L^ homozygous mutants showed a comparable degree of TA denervation as in the heterozygous mutant control (Supplementary Fig. 4C).

### Abnormal NMJ structure and function early in FUS mutants

Ultrastructural changes of NMJs in the TA muscle were also observed in the τ^ON^hFUS^P525L^ mice at p30 when morphological changes were first seen by light microscopy (Fig. 4c,d). At this early time point, mutant NMJs showed pre-synaptic abnormalities, including a significant (∼40%) reduction in the density of synaptic vesicles (depicted in yellow in Fig. 4c1, c2, d1 and d2) in the pre-synaptic terminals of the mutant (39.7±1.4 per μm^2^) compared with aged-matched WT controls (64.3±6.6 per μm^2^; Fig. 4c–e). However, the number of active zones—the main apparatus for synaptic vesicle release—was unchanged in the τ^ON^hFUS^P525L^ mice compared with the τ^ON^hFUS^WT^ control (2.4±0.2 per μm^−1^ and 2.6±0.2 per μm^−1^, respectively). In the axon terminals of the τ^ON^hFUS^P525L^ mutant, the number of morphologically normal mitochondria (6.3±2.0 per μm^2^) was significantly decreased compared with control terminals (34.1±10.1 per μm^2^; Fig. 4c,d, purple) and the remaining mitochondria appeared dilated and vacuolated with disorganized cristae and membranes (Fig. 4d and d1–2), comparable to that described in mutant SOD1 and TDP-43 transgenic models of ALS^40^. At the postsynaptic site, we also found evidence of muscle degeneration in the τ^ON^hFUS^P525L^ mice including abnormal mitochondria (Fig. 4d). Further, the sarcomere length in TA muscle of the τ^ON^hFUS^P525L^ mutant (1.83±0.2 μm) was significantly reduced compared with control fibres (3.04±0.05 μm; Fig. 4e). These data suggest that early synaptic changes in the τhFUS mutant mice lead to severe NMJ abnormalities and the consequent axonal detachment observed in our immunofluorescent studies.

To determine whether these ultrastructural changes are associated with physiological dysfunction at the NMJ, we used electromyography (EMG) to record spontaneous and evoked activity of TA MNs at the NMJ in the τ^ON^hFUS^P525L^ and WT control mice. Using a novel, *in vitro* sciatic nerve-hind-limb preparation, we stimulated the sciatic nerve for 1 s at 10, 50 and 100 Hz and recorded the evoked motor response from the TA muscle. We detected no difference in the motor response in the τ^ON^hFUS^P525L^ mice compared with controls at 2 weeks of age (Supplementary Fig. 5E,F), demonstrating no developmental abnormality in NMJ transmission. At 3 weeks of age, there was no significant difference in the average motor response between control and mutant mice with low frequency stimulation (10 Hz; Supplementary Fig. 5A,B). However, at 50 Hz there was a significant (∼45%) reduction of the motor response amplitude in the TA of mutant mice compared with controls (Fig. 4f,g), suggesting that mutant MNs cannot sustain reliable neurotransmission at the NMJs and exhibit abnormal signs of synaptic depression. At 100 Hz, both control and mutant mice exhibited synaptic depression as measured by the average amplitude of the motor response (Supplementary Fig. 5C,D), however, in contrast to the controls, the motor response in mutant TA MNs was often completely absent, indicating a failure of neurotransmission. As further evidence of dysfunction at the NMJ in the FUS mutant mice, we observed a significant increase in spontaneous activity in the τ^ON^hFUS^P525L^ mutants, a sign of active denervation of neurogenic origin (Fig. 4h,i). Although the frequency and amplitude of these events were variable between mutants, control animals did not exhibit any spontaneous events. Together, these results provide strong evidence that in τ^ON^hFUS^P525L^ mutant mice, functional abnormalities in neurotransmission at the NMJ precede gross morphological changes and motor axon withdrawal.

### Cell autonomous MN degeneration in FUS mutant mice

**Figure 5:**
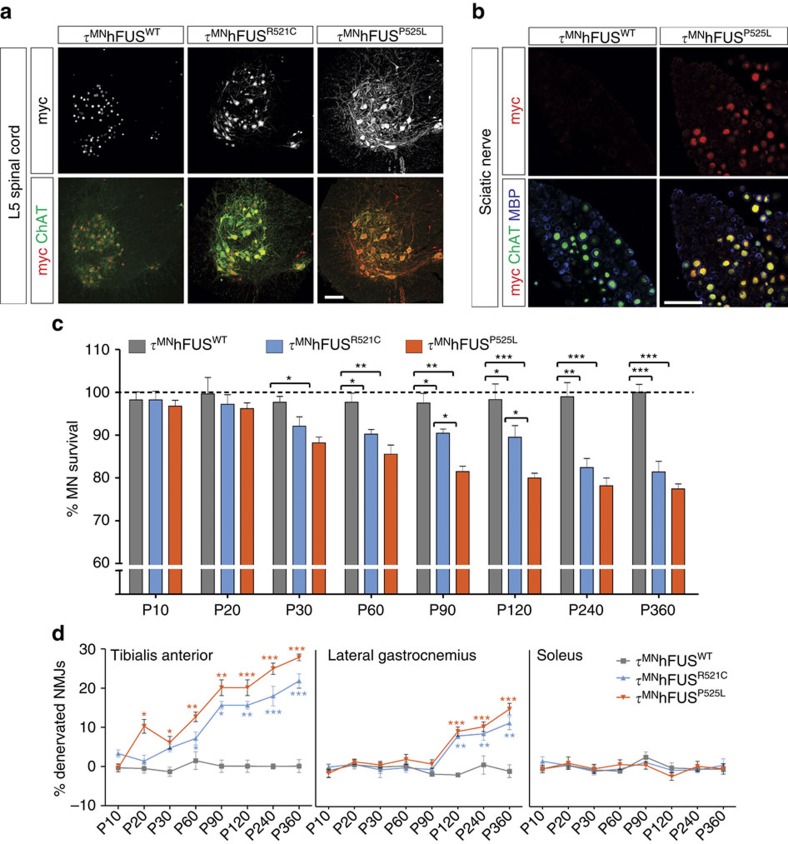
Motor neuron-selective expression of mutant FUS results in degeneration of MNs and denervation of fast muscles. (**a**) Conditional expression of myc-tagged hFUS proteins (grey, top) in cholinergic cells of τ^MN^hFUS^WT^, τ^MN^hFUS^R521C^ and τ^MN^hFUS^P525L^ animals. hFUS expression was restricted to cholinergic cells, as determined by the co-localization of myc (red, bottom) with ChAT (green, bottom). Scale bar, 100 μm. (**b**) Cross-sections of the sciatic nerves from τ^MN^hFUS^WT^ (left) and τ^MN^hFUS^P525L^ (right) animals stained with anti-myc (red), anti-ChAT (green) and anti-MBP (blue) antibodies. Co-localization of hFUS and ChAT in motor axons (yellow) was observed in τ^MN^hFUS^P525L^ but not in τ^MN^hFUS^WT^ animals. Scale bar, 20 μm. (**c**) MN survival in τ^MN^hFUS^WT^ (light grey), τ^MN^hFUS^R521C^ (light blue) and τ^MN^hFUS^P525L^ (orange) animals calculated from the number of MNs in the L5 spinal cord normalized to that of non-transgenic wild-type controls (expressed as a percentage). (**d**) Percentages of denervated NMJs in the tibialis anterior, gastrocnemius and soleus muscles of τ^MN^hFUS^WT^ (light grey), τ^MN^hFUS^R521C^ (light blue) and τ^MN^hFUS^P525L^ (orange) animals normalized to non-transgenic wild-type controls. (For **c** and **d**: *P<0.05, **P<0.01 and ***P<0.001 using one-way analysis of variance at each time point with Bonferroni's *post hoc* test. Error bars represent s.e.m. *N*=4 for all genotypes.)

To test whether expression of mutant FUS in MNs alone is sufficient to cause MN degeneration, we used a ChAT-Cre deleter strain crossed to the conditional τ^OFF^hFUS^WT^, hFUS^R521C^ and hFUS^P525L^ lines to express each hFUS isoform selectively in MNs (Fig. 5a). We refer to this series of mice as the τ^MN^ lines. Immunohistochemical analysis of the τ^MN^hFUS^R521C^ and τ^MN^hFUS^P525L^ spinal cords revealed marked mislocalization of mutant hFUS to the cytoplasm, dendrites and axons of MNs (Fig. 5a,b). In contrast, WT hFUS remained localized to MN nuclei in the τ^MN^hFUS^WT^ mice, similar to what we observed in the τ^ON^hFUS^WT^ controls (Figs 5a and 2b). Immunohistochemical (Fig. 5b) and western blot analysis (Fig. 1d) of sciatic nerve also detected myc-hFUS^R521C^ and -hFUS^P525L^, but not -hFUS^WT^ in the periphery, confirming that ALS mutant FUS is selectively mislocalized to motor axons in the τ^MN^ mutants.

To determine whether the selective expression of ALS mutant FUS in MNs caused cell autonomous degeneration *in vivo*, we counted all ChAT+ MNs at L5 (Fig. 5c). At p10, there was no significant difference in the number of L5 MNs in the τ^MN^ mutants compared with the τ^MN^hFUS^WT^ and WT controls. At p30, we observed significant MN loss in the τ^MN^hFUS^P525L^ (11.8%, Fig. 5c) mutant mice compared with both τ^MN^hFUS^WT^ and control animals. Significant degeneration was first noted in the τ^MN^hFUS^R521C^ at p60 when 10% of all L5 MNs were lost (Fig. 5c). This degeneration progressed in both ALS mutants until approximately p240, and measured at p360 there were 18.6% and 22.6% fewer MNs overall in the L5 segment of τ^MN^hFUS^R521C^ and τ^MN^hFUS^P525L^ mutants, respectively, compared with age-matched controls (Fig. 5c).

MN loss in the τ^MN^hFUS mutants was also associated with significant (∼10%) denervation of the TA NMJs, first observed at p20 of in the τ^MN^hFUS^P525L^ mutants (Fig. 5d) before any detectable MN loss at L5 (Fig. 5c), and at p60 in the τ^MN^hFUS^R521C^ mice (Fig. 5d). Denervation of the TA muscle progressed with age, and at p360 ∼30% and ∼25% of NMJs were denervated in the τ^MN^hFUS^P525L^ and τ^MN^hFUS^R521C^ mutants, respectively (Fig. 5d). Significant denervation was first noted much later (p120) in the GS in both the τ^MN^hFUS^P525L^ (∼13%) and τ^MN^hFUS^R521C^ (∼10%) mice (Fig. 5d), but even at p360, no significant denervation was noted in the Sol in any of the genotypes we analysed. Together, these data demonstrate that selective expression of mutant hFUS in MNs is sufficient to cause muscle denervation and MN degeneration by cell autonomous mechanisms, preferentially affecting MN subtypes most vulnerable in ALS.

### FUS loss of function alone does not cause MN degeneration

**Figure 6:**
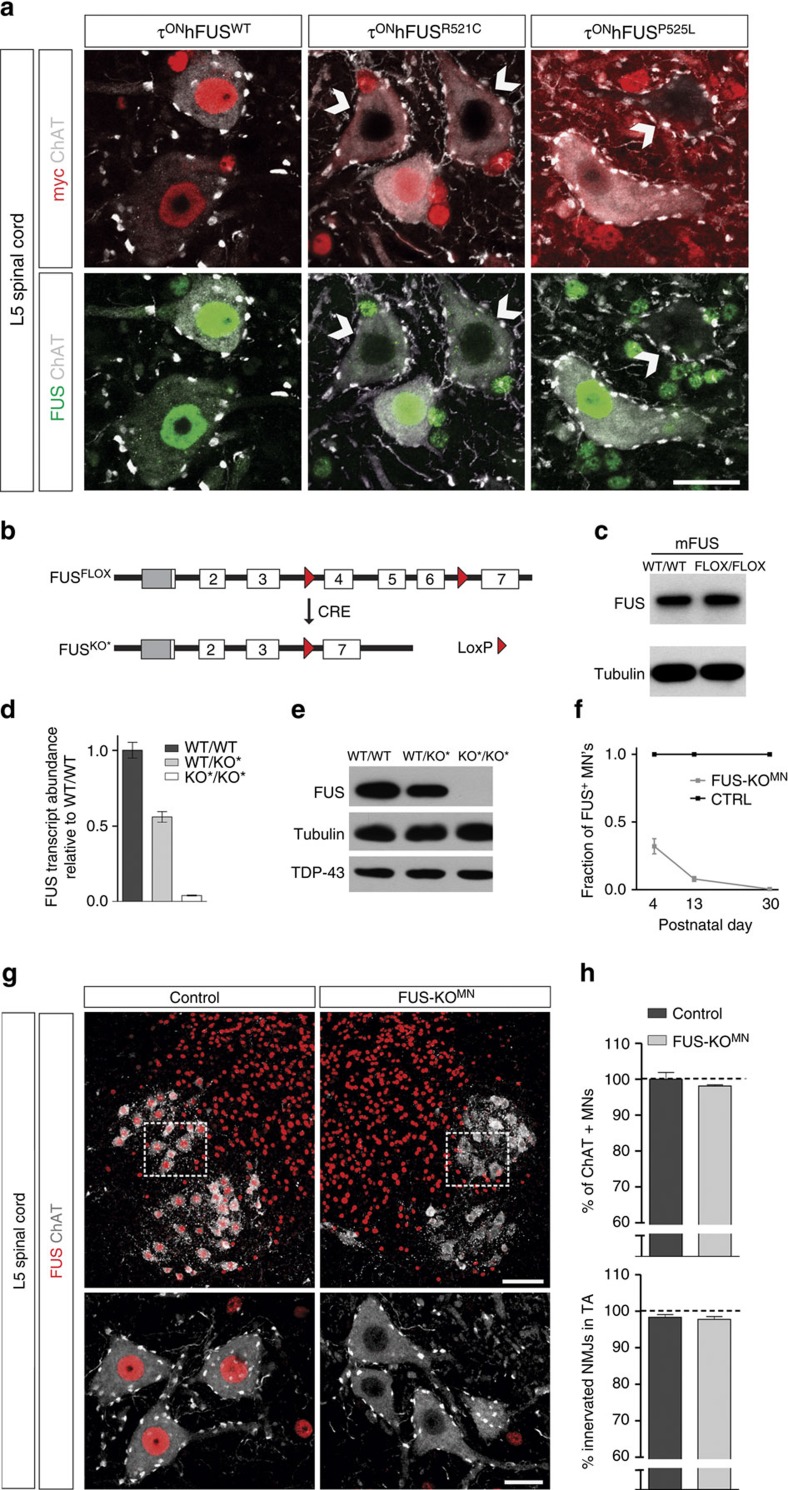
Motor neuron-selective deletion of *FUS* does not cause MN degeneration. (**a**) MNs of τ^ON^hFUS^WT^ (left), τ^ON^hFUS^R521C^ (centre) and τ^ON^hFUS^P525L^ (right) animals immunostained with anti-myc (red, top) and anti-FUS (green, bottom) antibodies. White arrows indicate cells with no nuclear staining for myc-hFUS (red) and endogenous mFUS (green). Scale bar, 25 μm. (**b**) Illustration of the conditional *FUS* allele (FUS^FLOX^) and the null *FUS* allele (FUS^KO*^). In the FUS^FLOX^ allele, exons 4–6 of murine *FUS* are flanked by LoxP (red triangles) recombination sites. Cre-dependent excision of exons 4–6 produces the FUS^KO*^ allele. (**c**) Western blot analysis of total brain extracts with anti-FUS antibody in p10 wild-type controls (WT/WT) and animals homozygous for the conditional *FUS* allele (FLOX/FLOX). (**d**) Using primers specific to exons 12–14, in p0 total brain extracts, *FUS* transcript abundance in *FUS* null (*FUS*^KO*/KO*^, light grey) was 4.1±0.3% relative to wild-type controls (dark grey) animals. Similar results (6.7±0.8%) were obtained using primers specific to exons 1–3 (*N*=3). (**e**) Western blot analysis of total brain extracts from p0 wild-type (WT/WT), heterozygous FUS null (WT/KO*) and homozygous FUS null (KO*/KO*). No protein products were observed in FUS^KO*/KO*^ animals using N-terminal anti-FUS antibody (ab84078). Similar results were obtained using antibodies raised to the middle and the C-terminus of the FUS protein (Supplementary Fig. 4D). (**f**) Quantification of the fraction of MNs (ChAT+) that were also FUS+ in control (black) and FUS-KO^MN^ (grey) animals. The majority of MNs (67.1±5.6%) were already immunonegative for FUS by p4 and by p30 the elimination of FUS from MNs was complete (99.5±0.3%). *N*=3. (**g**) Immunostaining of L5 spinal cords from p360 control (left) and FUS-KO^MN^ (right) animals for FUS (red) and ChAT (white). Boxed area is reproduced at higher magnification in the bottom panels. Scale bar, 100 μm (top), 25 μm (bottom). (**h**) Percentage of MNs in the L5 spinal cord normalized to control (top) and the percentages of innervated NMJs in the TA muscle (bottom) in p360 control (dark grey) and FUS-KO^MN^ (light grey) animals. *N*=4. (For **f**–**h**: Control=FUS^FLOX/**WT**^;ChAT-Cre^+/−^ and FUS-KO^MN^=FUS^FLOX/**KO**^;ChAT-Cre^+/−^).

In ALS-FUS, mutant hFUS may cause MN degeneration either by acting as a dominant-negative to inhibit normal FUS activity or through a novel, toxic gain of function. A loss-of-function mechanism would be consistent with our observation that mutant hFUS as well as endogenous mFUS was excluded from the nucleus in a significant subset of MNs in the τ^ON^hFUS mutants (Fig. 6a), as reported in ALS-FUS patients^24^. At p120, FUS exclusion was observed in ∼5–10% of L5 MNs in the τ^ON^hFUS^R521C^ and τ^ON^hFUS^P525L^ mutants, and rarely in the τ^ON^hFUS^WT^ and WT controls. Nuclear exclusion of hFUS and endogenous mFUS was also observed in a proportion of MNs in the τ^MN^hFUS mutant mice (Supplementary Fig. 2C).

To explore the role of FUS loss of function on MN degeneration in the τ^ON^hFUS mutants, we generated a novel, conditional FUS knockout (FUS^FLOX^) allele (Fig. 6b) to overcome the perinatal lethality of the constitutive FUS knockout mutation^41^. FUS transcript (by RT–qPCR (*N*=3)) and protein (Fig. 6c) levels in the postnatal brain were equivalent in the FUS^FLOX/FLOX^ and WT control, and homozygous FUS^FLOX/FLOX^ and FUS^FLOX/KO^ mutants showed normal postnatal survival. Together, these findings demonstrate that before recombination, the conditional FUS^FLOX^ allele is fully functional and able to rescue the perinatal lethality of FUS deficiency. We then crossed the FUS^FLOX^ mice to a germline Cre deleter strain to generate a constitutive FUS knockout allele (FUS^KO*^; Fig. 6b) and found that homozygous FUS^KO*/KO*^ mutants die at birth and express no detectable level of FUS transcript or protein at p0 (Fig. 6d,e). These data demonstrate that upon recombination, FUS^FLOX^ generates a null (FUS^KO*^) allele.

To determine whether FUS deficiency in MNs causes neurodegeneration, we used *ChAT*-Cre to selectively eliminate FUS from MNs by generating mice heterozygous for the FUS^FLOX^, FUS^KO^ and ChAT-Cre alleles (referred to as FUS-KO^MN^ mutants). We reasoned that if ALS mutant FUS acts as a dominant negative to cause MN degeneration in the τhFUS mutants and ALS-FUS patients, then the complete deletion of FUS from MNs would result in at least comparable if not more severe loss of MNs in the FUS-KO^MN^ mice. Analysis of the FUS-KO^MN^ mutants revealed selective and nearly complete elimination of FUS from MNs in the ventral horn of the L5 spinal cord by the end of the second postnatal week (Fig. 6f,g). Remarkably, despite this cell autonomous loss of FUS, no appreciable MN degeneration was observed in the FUS-KO^MN^ mutant compared with controls even at 1 year of age (p360), as measured by the number of L5 MN somata and the extent of denervation of NMJs in the TA muscle (Fig. 6h).

**Figure 7:**
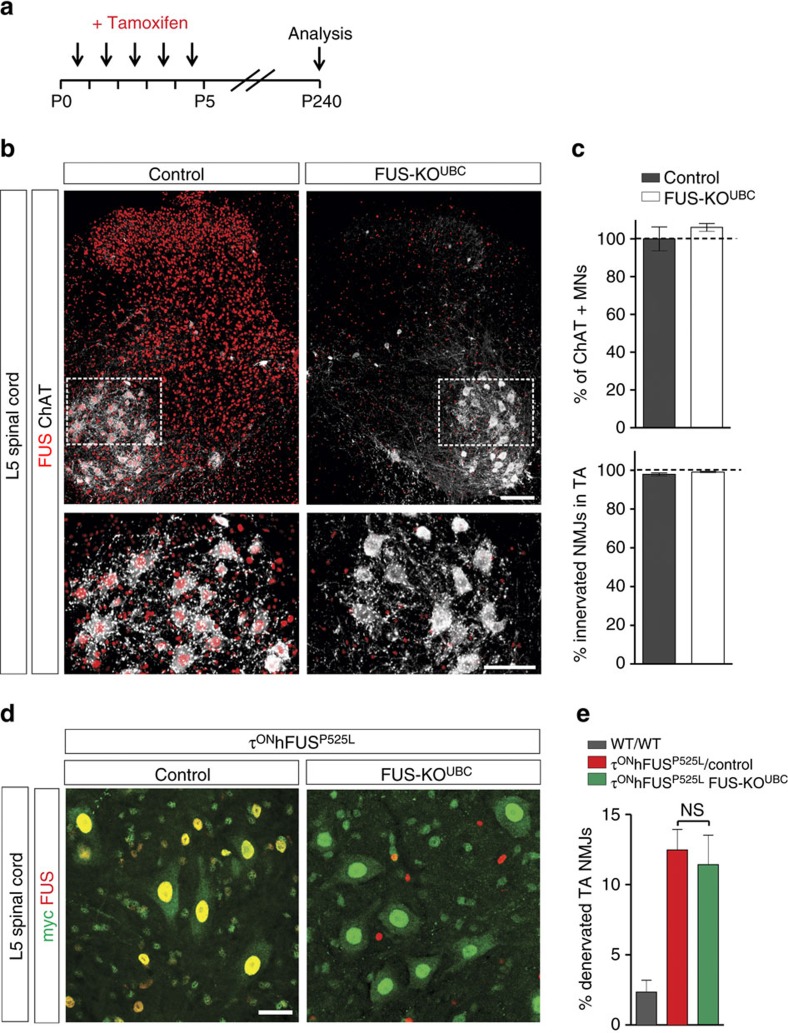
Widespread postnatal deletion of *FUS* does not cause MN degeneration. (**a**) Experimental paradigm for early postnatal induction of CreERT2 activity. Mice were treated with tamoxifen at postnatal days 1–5 and were analysed at p240. (**b**) L5 spinal cords of p240 control (FUS^FLOX/**WT**^; UBC-CreERT2^+/−^, left) and FUS-KO^UBC^ (FUS^FLOX/**KO**^; UBC-CreERT2^+/−^, right) animals immunostained for FUS (red) and ChAT (white). Outlined areas of top panels are reproduced at higher magnification in the bottom panels. Loss of FUS immunoreactivity was observed in 96.0±3.8% of NeuN-IR neurons and 83.0±4.8% of NeuN-negative cells. *N*=3. Scale bar, 200 μm (top), 50 μm (bottom). (**c**) The numbers of MNs in the L5 spinal cord normalized to control (top) and the percentage of innervated NMJs in the tibialis anterior (TA) muscle (bottom) in p240 control (dark grey) and FUS-KO^UBC^ (white) animals. *N*=3. (**d**) Representative images of L5 MNs in τ^ON^hFUS^P525L^/control (left) and τ^ON^hFUS^P525L^/FUS-KO^UBC^ animals (right). (**e**) Percentage of denervated NMJ's in the TA muscle of τ^ON^hFUS^P525L^/control (red) and τ^ON^hFUS^P525L^/FUS-KO^UBC^ (green) animals (*N*=4 for all groups. For **d** and **e**, the genotypes of the τ^ON^hFUS^P525L^/control and the τ^ON^hFUS^P525L^/FUS-KO^UBC^ animals were τ^ON^hFUS^P525L^;FUS^FLOX/**WT**^;UBC-CreERT2^+/−^ and τ^ON^hFUS^P525L^;FUS^FLOX/**KO**^;UBC-CreERT2^+/−^, respectively.)

As a further test of whether FUS loss of function could cause MN degeneration, we used a ubiquitously expressed, tamoxifen-inducible Cre deleter (UBC-CreERT2 (ref. 42)) to induce widespread, postnatal elimination of FUS in the central nervous system (see Methods for details). Early postnatal FUS^FLOX^ recombination (Fig. 7a) led to the loss of FUS immunoreactivity in a large majority of all neuronal and non-neuronal cells in the L5 spinal cord (shown at p240, Fig. 7b). Again, despite this early and efficient elimination of FUS expression, no significant loss of L5 MNs or denervation of the TA muscle was detected at p240 (Fig. 7c), well after MN degeneration was observed in the τ^ON^hFUS^R521C^ and τ^ON^hFUS^P525L^ mice. Together, these data demonstrate that postnatal FUS expression is not required for the long-term survival of MNs *in vivo* and that FUS elimination does not reproduce the MN degenerative phenotype observed in the τhFUS mutants.

### FUS toxicity is unrelated to endogenous mouse FUS expression

The absence of a MN phenotype in the postnatal FUS knockout mutant leads to the conclusion that MN degeneration in the τhFUS mutant mice is caused by a toxic gain of function. Although mutant hFUS protein in the τ^ON^ mice is expressed far below the levels of endogenous mFUS (Fig. 1d), MN toxicity may be a consequence of excess of FUS activity. Our finding that the ALS-related mutations increase the stability and steady-state level of hFUS (Fig. 1d,f) is consistent with the idea that MN toxicity is a function of increased FUS expression. Alternatively, mutant hFUS may interact with mFUS to seed the formation of toxic aggregates^21^,^43^,^44^. To test these possible gain-of-function mechanisms, we generated inducible FUS knockout (*FUS*^FLOX/KO^; UBC-CreERT2) mice that were also heterozygous for the τ^ON^hFUS^P525L^ allele to determine whether the elimination of endogenous mFUS would delay the onset of MN degeneration in the τ^ON^hFUS mutant. Postnatal treatment of these mutants with tamoxifen (Fig. 7a) again resulted in elimination of endogenous mFUS from a large majority (∼90.0%) of NeuN+ L5 spinal neurons (Fig. 7d). We used denervation of the TA muscle at p60 as a measure of disease initiation to determine whether this loss of endogenous FUS had any effect on the onset of the τ^ON^hFUS^P525L^ MN phenotype. Despite the widespread elimination of mFUS, we found no difference in the degree of TA denervation in this tamoxifen-treated mutant (∼12%) compared with littermate controls (∼13%) in which mFUS was expressed in all cells (Fig. 7e). These data provide functional evidence that the toxicity of mutant hFUS is not a consequence of an excess of total FUS. Consistent with our finding that mutant hFUS and endogenous mFUS do not interact physically (Fig. 1e), these data also demonstrates that mutant hFUS toxicity does not depend on an interaction between mutant and WT protein.

Together, our data demonstrate that the selective toxicity of mutant hFUS in the τ^ON^ mice is not due to an excess of total FUS or the mutation-related increase in hFUS stability, but rather to a toxic gain-of-function specific to the ALS-associated mutant protein.

## Discussion

Advances in ALS genetics have led to the identification of a multitude of genes in which mutations cause selective MN degeneration. The discovery of each new ALS gene presents an opportunity to generate novel models with which to explore mechanisms of disease and to identify therapeutic targets shared between distinct, familial and sporadic forms of ALS. ALS-FUS is unusual in that a wide variety of clinical phenotypes are associated with FUS mutations, ranging from common presentations of the disease to the most aggressive, juvenile-onset forms of ALS reported. Here using a series of novel mouse models of ALS-FUS, we demonstrate that FUS loss of function is not sufficient to cause the selective and progressive loss of MNs associated with ALS mutant FUS and that the toxicity conferred by ALS mutations on FUS does not involve an excess of FUS activity or require the interaction of mutant and WT FUS.

FUS expression is highly regulated and existing animal and cellular models have shown that an excess of WT FUS activity is toxic^14^, although not necessarily in a way that is relevant to ALS. We modelled FUS-ALS by generating a series of targeted transgenic animals in which equivalent levels of exogenous hFUS transcript are stably expressed from a defined locus in a consistent pattern, at or below endogenous FUS levels so as to avoid any variance in the lines that may influence the MN phenotype. Analysis of animals expressing mutant hFUS selectively from spinal MNs (τ^MN^) or more broadly from all τ-expressing neurons and non-neuronal cells (τ^ON^) revealed progressive MN degeneration that selectively involves spinal MNs and spares neuronal populations normally unaffected in ALS, including oculo-MNs and other long projection neurons like muscle spindle (*I*_A_) afferents in the dorsal root ganglia. We also observed the preferential involvement of fast versus slow twitch motor units in our models, demonstrating the selective loss of specific, functional subtypes of spinal MNs most vulnerable in patients and SOD1 mutant mice^37^. In contrast, no MN loss or muscle denervation was observed in the heterozygous or homozygous hFUS^WT^ animals, demonstrating that the MN phenotype we describe is dependent on the ALS-causing mutation in hFUS. The molecular and cellular selectivity of mutant FUS toxicity in this system demonstrates the relevance of the τ^ON^-hFUS mouse model to FUS-ALS. However, the selective involvement of a specific subset of MNs—while sufficient to cause significant hind-limb weakness—does not influence the lifespan of these mutants. Although this is in some respects a limitation of the model, the early onset and steady progression of MN degeneration, together with the specific, qualitative aspects of the MN phenotype make the τ^ON^-hFUS mutants a highly ALS-relevant system in which to explore disease mechanisms and to test potential therapeutics.

The conditional nature of the τ-hFUS alleles allowed us to explore whether cell autonomous and non-autonomous mechanisms contribute to mutant FUS-dependent MN degeneration, as in SOD1 mice^45^,^46^,^47^,^48^. We found that selective expression of mutant hFUS in MNs caused MN degeneration with the same selectivity as in the τ^ON^ mutants. These data do not argue that ALS-FUS is a cell autonomous disease, only that expression of mutant FUS in MNs alone is *sufficient* to cause MN loss. Even in the τ^MN^ mutants, evidence of astrocytosis and microgliosis suggests that secondary, non-autonomous mechanisms may also contribute to the MN phenotype in this model. Comparison of the τ^MN^- and the corresponding τ^ON^-hFUS mutant (Supplementary Fig. 3D) did not reveal a significant non-autonomous effect in the hFUS^R521C^ model, but in the hFUS^P525L^ mice the extent of MN loss and the degree of denervation was significantly greater in the τ^ON^ versus τ^MN^ mutant, but only after p240. These studies show that cell non-autonomous expression of mutant hFUS^P525L^ had no effect on the onset of MN degeneration, but did accelerate disease progression in this mouse model of ALS-FUS. The specific cell type(s) involved in the cell non-autonomous mechanisms of disease progression in the τ^ON^ mice remains to be determined, but the predominant expression of *MAPT* in neurons and oligodendroglia (Supplementary Fig. 3C) implicates these MN partners in FUS-mediated MN degeneration.

MN degeneration in the τ-hFUS mutants progresses slowly, but the first evidence of pathology is detected remarkably early in the most vulnerable motor units of the τ^ON^hFUS^P525L^ mice. By the third postnatal week, structural and functional evidence of synaptic failure at the NMJ is first observed. Pre- and postsynaptic changes in the ultrastructure of the NMJ—including abnormal mitochondria and reduced synaptic vesicle density—are associated with electrophysiological evidence of active denervation of the TA muscle, including increased spontaneous activity and sensitivity to high-frequency stimulation. These early changes at the NMJ anticipate the retraction of the motor axon ∼10 days later in the τ^ON^hFUS^P525L^ mouse and precede loss of MN somata. These data are consistent with a model of axonal ‘die-back' described in the SOD1 mutant by which degenerative changes at the NMJ occur in advance of neuronal loss in distinct subpopulations of MNs^49^. The mechanism that underlies these early physiological and morphological changes at the NMJ is not known, but overexpression of mutant FUS has been reported to impair axonal transport^50^,^51^,^52^. The reported roles of FUS in mRNA transport and local translation^17^ are perturbed by ALS-associated mutations^53^,^54^, suggesting that—as with mutant TDP-43 (ref. 20)—mutant FUS may impair the transport of mRNA to the periphery and decrease axonal translation required for axonal maintenance^55^.

The discovery of novel ALS genes leads first to the question of whether causal mutations result in a loss of function of the encoded protein or a gain of toxic properties, related or not to its normal activity. Structural and functional similarities between TDP-43, FUS and other ALS-associated RNA-binding proteins encouraged the view that the role of these proteins in MN disease relates to the normal RNA processing functions of these regulatory factors. Physical interaction between FUS and TDP-43 as well as evidence of their shared role in genetic pathways required for survival and motor function^56^,^57^ further implicated the normal activities of these proteins in MN survival in ALS. These early observations led to the suggestion that mutant FUS acts as a dominant negative to interfere with the normal activity of the WT FUS protein to cause a partial or complete loss of function, leading to MN degeneration in ALS-FUS. Our data demonstrating that postnatal FUS elimination has no effect on MN survival even in the most vulnerable MN pools provide unequivocal evidence that FUS loss-of-function alone does not underlie MN degeneration in ALS.

In ALS patients, FUS^P525L^ is associated with juvenile-onset and rapidly progressive MN disease. In the τ^ON^hFUS mice, this allele is also more pathogenic than hFUS^R521C^ both in terms of onset and progression of the MN degeneration. This provides further evidence that the τ-hFUS mice reliably model key aspects of ALS-FUS. However, the basis of this enhanced toxicity is not clear. In the τ-hFUS^P525L^ mice, mutant FUS is localized to the cytoplasm to a much greater extent than hFUS^R521C^, and the more pronounced MN phenotype is consistent with previous studies that correlate toxicity with the degree of cytoplasmic mislocalization of FUS^58^. However, although the accumulation of cytoplasmic FUS correlates with the severity of the MN phenotype in our model, we find no evidence that this is associated with the formation of large cytoplasmic inclusions characteristic of ALS-FUS.

Toxicity in the τ-hFUS mice was also associated with an increase in the steady-state level of mutant hFUS protein relative to the hFUS^WT^ control, which does not reflect a difference in the levels of hFUS mRNA in the three τ^ON^-hFUS mice, but an increase in the stability of hFUS conferred by ALS-causing mutations—in agreement with a recent *in vitro* study^59^. However, the selective toxicity of mutant hFUS does not appear to be a quantitative/dose-dependent effect in this system as doubling of hFUS^WT^ levels in the homozygous τ^ON^ mutant does not result in MN degeneration, and twice the level of mutant hFUS^P525L^ does not exacerbate the MN phenotype. Rather, these data support a mechanism by which ALS-related mutations confer qualitative changes on hFUS that underlie the toxic gain of function.

Insight into the mechanism by which mutant FUS causes MN degeneration in the τ^ON^ mice comes from our finding that the postnatal elimination of the endogenous FUS in our most severe hFUS^P525L^ mutant had no effect on the initiation and early progression of MN loss. These data argue that mutant FUS toxicity does not involve an excess of FUS activity, as suggested by the finding that mutations in the 3′UTR of FUS^13^ cause ALS by perturbing the autoregulation of FUS expression^60^ and increasing FUS^WT^ levels^13^, and by previous studies showing that overexpression of FUS^WT^ is toxic to neurons. Our data also demonstrate that the toxicity of mutant FUS does not depend on a physical or functional interaction with the much more^14^,^61^ abundant endogenous FUS protein, arguing against the ‘seeding' hypothesis of FUS toxicity by which mutant hFUS accelerates the aggregation of mFUS^WT^, resulting in selective toxicity to MNs.

In light of our conclusion that the activity of FUS is not required for the long-term survival of MNs, the question remains then as to how the structural and functional similarities between FUS-, TDP-43- and other ALS-associated hnRNP's relate to their role in the disease. One intriguing idea is that these ALS proteins all contain low-complexity ‘prion-like' sequences (and possibly other domains^62^) that are not only a key determinant of their normal function, but also of their role in the pathogenesis of ALS. These domains appear to mediate the movement of these regulatory proteins in and out of subcellular, RNA-containing structures like stress granules^43^,^44^ and permit reversible and regulated^44^,^63^,^64^ interactions with a large variety of partner proteins. Under pathological conditions, these same regions may mediate the irreversible formation of abnormal aggregates that are selectively toxic to subpopulations of neurons. The natural propensity of these proteins to anneal leads to a model of disease in which the low probability of WT protein to form these toxic aggregates is increased by disease-causing mutations, leading to the onset of neurodegeneration^7^. Although our data does not support the seeding of endogenous FUS by the mutant, aberrant interactions of mutant hFUS with other factors could underlie its toxicity, and the ALS-FUS models we present offer a highly disease-relevant*, in vivo* system in which to explore these interactions and to elucidate mutant FUS-dependent mechanisms of disease.

## Methods

### Generation of mice and mouse genetics

All procedures were performed in accordance with the National Institutes of Health Guidelines on the Care and Use of Animals and approved by the Institutional Animal Care and Use Committee at Columbia University.

The τ^OFF^hFUS mouse lines were generated using a vector modified from that designed to target the mouse *MAPT* (tau) genomic locus by homologous recombination^65^. This targeting vector was modified to include a LoxP-flanked transcriptional ‘stop' sequence (poly-A^3^) followed by a myc-tagged human FUS cDNA (hFUS^WT^, hFUS^R521C^ or hFUS^P525L^ generously provided by Brian McCabe).

The FUS^FLOX^ mouse line in which FUS exons 4–6 are flanked by LoxP sites was created from ES cells obtained from the International Knockout Mouse Consortium Project: 84575.

### Muscle innervation analysis

Cryosections of muscle (30 μm) were stained with antibodies against VAChT to identify the pre-synaptic terminal, and tetramethylrhodamine-conjugated α-BTX (Invitrogen) to detect post-synaptic acetylcholine receptors. Images were acquired using Zeiss Pascal LSM 510 confocal microscope using a × 10 objective. Percentage (%)NMJ innervation was determined by dividing the total number of areas of overlap between VAChT and α-BTX signals (total number innervated endplates) by the number of areas α-BTX signal (total number of endplates).

### MN counts

The lateral motor column of lumbar spinal segment L5 was imaged as a z-series of confocal optical sections at a × 20 magnification (z-step of 1.5 μm). MNs labelled with an anti-ChAT antibody were counted by outlining the cell body in the confocal plane of the nucleolus using ImageJ.

### Electron microscopy

Animals were deeply anaesthetized using ketamine (100 mg kg^−1^) and xylazine (10 mg kg^−1^) and transcardially perfused with 0.1 M PBS followed by 2.5% glutaraldehyde and 4% paraformaldehyde (EMS) in PBS. TA muscles were post-fixed at 4 °C overnight and sectioned using a vibrating blade microtome (200 μm; Leica VT 1000S) and processed for reduced osmium tetroxide-thiocarbohydrazide—osmium (ROTO) method^66^. After dehydration and resin infiltration (EPON 812; EMS), samples were cured at 70 °C for 3 days. Blocks were sectioned in a UC7 Ultramicrotome (Leica) with a 35° Diamond knife (Diatome). Ultra-thin serial sections (40 nm) were collected on Kapton^66^, post-stained (1% uranyl acetate, 5 min; 2% lead citrate, 1 min), mounted in silicon wafers (Universal Wafers) and carbon coated (custom built coater). Backscattered images were collected using ATLAS (FIBICS) at 3 nm pixel resolution (3 ms dwell) in a SIGMA FESEM electron microscope (Zeiss).

### NMJ functional assays

Mice were terminally anaesthetized with isoflurane followed by decapitation. The M-response in TA muscles was assessed in an isolated sciatic nerve-hind-limb preparation *in vitro.* Muscles were perfused with oxygenated (95%O_2_+5%CO_2_) artificial cerebrospinal fluid (CSF) (12.83 mM NaCl, 0.4 mM KCl, 0.05 mM NaH_2_PO_4_, 2.1 mM NaHCO_3_, 1.5 mM CaCl_2_, 1 mM MgSO_4_, 30 mM glucose). A suction stimulating electrode was placed on the sciatic nerve and EMG recordings were obtained using a concentric needle electrode (P12CEA3, Microprobes) placed in the belly of the muscle. The maximum M-response was obtained following stimulation of the sciatic nerve for 0.2 ms at supramaximal stimulus intensity. The signal was amplified ( × 1,000; acquired by MultiClamp 700B, Molecular Devices), filtered (0.1–3 kHz) and digitized for offline analysis.

Spontaneous muscle activity was recorded for 60 s immediately after placement of the EMG electrode. A two Standard Deviations (2s.d.) window discriminator of the peak-to-peak baseline signal (calculated from 5 different and random time points during EMG acquisition) was used to calculate spontaneous events above the baseline recording.

Subsequently, a train of stimuli was delivered at 10, 50 and 100 Hz to the sciatic nerve for 1 s duration. The change of the M-response (measured peak-to-peak) at different frequencies was calculated and expressed as a percentage change from the first response for each frequency.

### Statistical analysis

For all statistical tests, Graph Pad Prism 6 software was used. Statistical analysis of mean differences between groups was performed using either one- or two-way analysis of variance, followed by a Bonferroni *post hoc* analysis or Fisher's least significant difference (LSD) test depending on the number of variables and time points in each experiment. The statistical tests, *P* values and *n* values are indicated in figure legends.

See Supplementary Methods for details.

## Additional information

**How to cite this article:** Sharma, A. *et al*. ALS-associated mutant FUS induces selective motor neuron degeneration through toxic gain of function. *Nat. Commun.* 7:10465 doi: 10.1038/ncomms10465 (2016).

## Supplementary Material

Supplementary Information Supplementary Figures 1-6, Supplementary Tables 1-2, Supplementary Methods and Supplementary References.
