# Supplementary material for: ALS-associated mutant FUS induces selective motor neuron degeneration through toxic gain of function
Source: Nat Commun. 2016 Feb 4;7:10465. doi: 10.1038/ncomms10465 (PMC4742863; doi:10.1038/ncomms10465)
Supplement: Supplementary Information — Supplementary Figures 1-6, Supplementary Tables 1-2, Supplementary Methods and Supplementary References. [file ncomms10465-s1.pdf]

Supplementary Figures

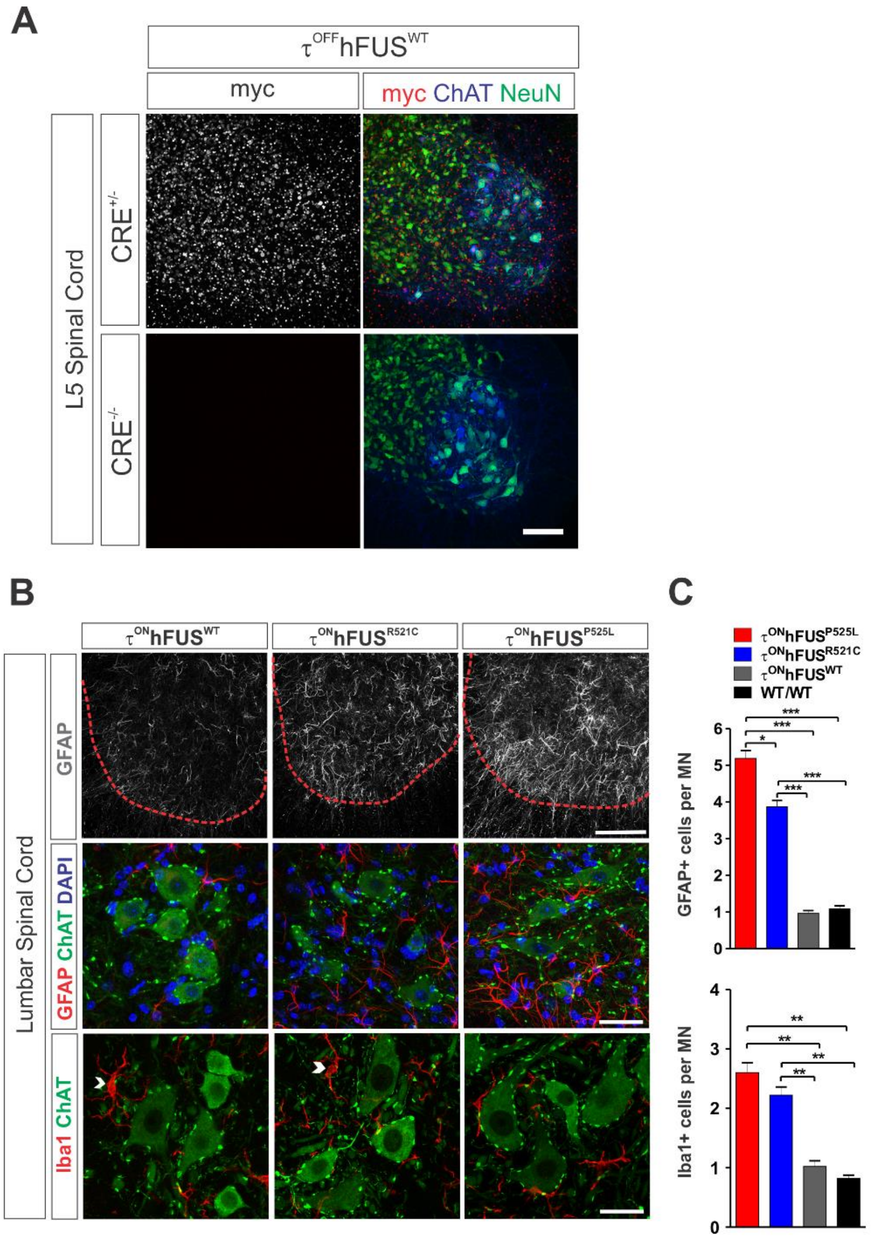

Supplementary Figure 1

(A) In the absence of Cre-recombinase there is no expression of myc-tagged hFUS from the τ^{OFF}hFUS *MAPT* locus. Scale bar = 100µm.

(B) Expression of the inflammatory markers GFAP (gray, top panel; red, middle panel) and Iba1 (red, bottom panel). Scale bar = 100µm (top panel) and 40µm (middle and bottom panels).

(C) The number of GFAP-positive (top) and Iba1-positive (bottom) cells in 100µm radial distance from lumbar MNs (50 MNs counted).

(All genotypes are p120, N=4. For B and D: *P < 0.05, **P < 0.01 and ***P < 0.001 using one-way ANOVA with Bonferroni's post hoc test. Error bars represent SEM.**)**

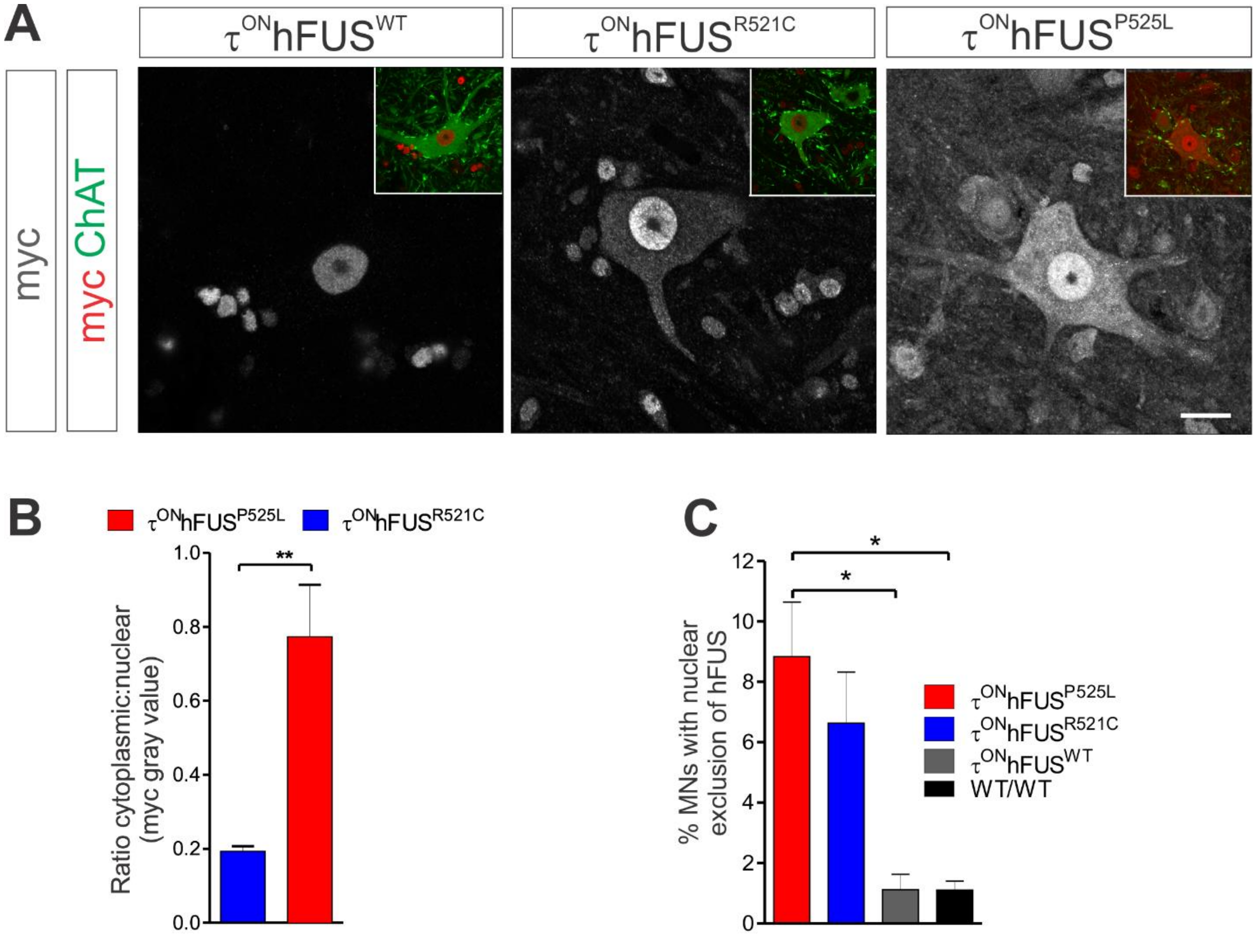

Supplementary Figure 2

(A) Representative confocal images showing the degree of cytoplasmic mislocalization of hFUS protein (gray) in ChAT positive MNs (inset, green). Scale bar = 40µm

(B) Ratios of cytoplasmic to nuclear gray values in myc-stained MN's of τ^{ON}hFUS^{R521C} (blue) and τ^{ON}hFUS^{P525L} (red) animals. All animals were p30, N=10 MNs from 3 animals for each genotype. **P<0.01. Statistical significance was calculated using a t-test.

(C) Percentage of total L5 MN's showing the absence of nuclear hFUS in τ^{ON}hFUS^{P525L} (red) and τ^{ON}hFUS^{R521C} (blue) compared to τ^{ON}hFUS^{WT} (gray) and control (black) mice. All animals were p90, N= 3 animals for each genotype. **P<0.01. Statistical significance was calculated using a t-test.

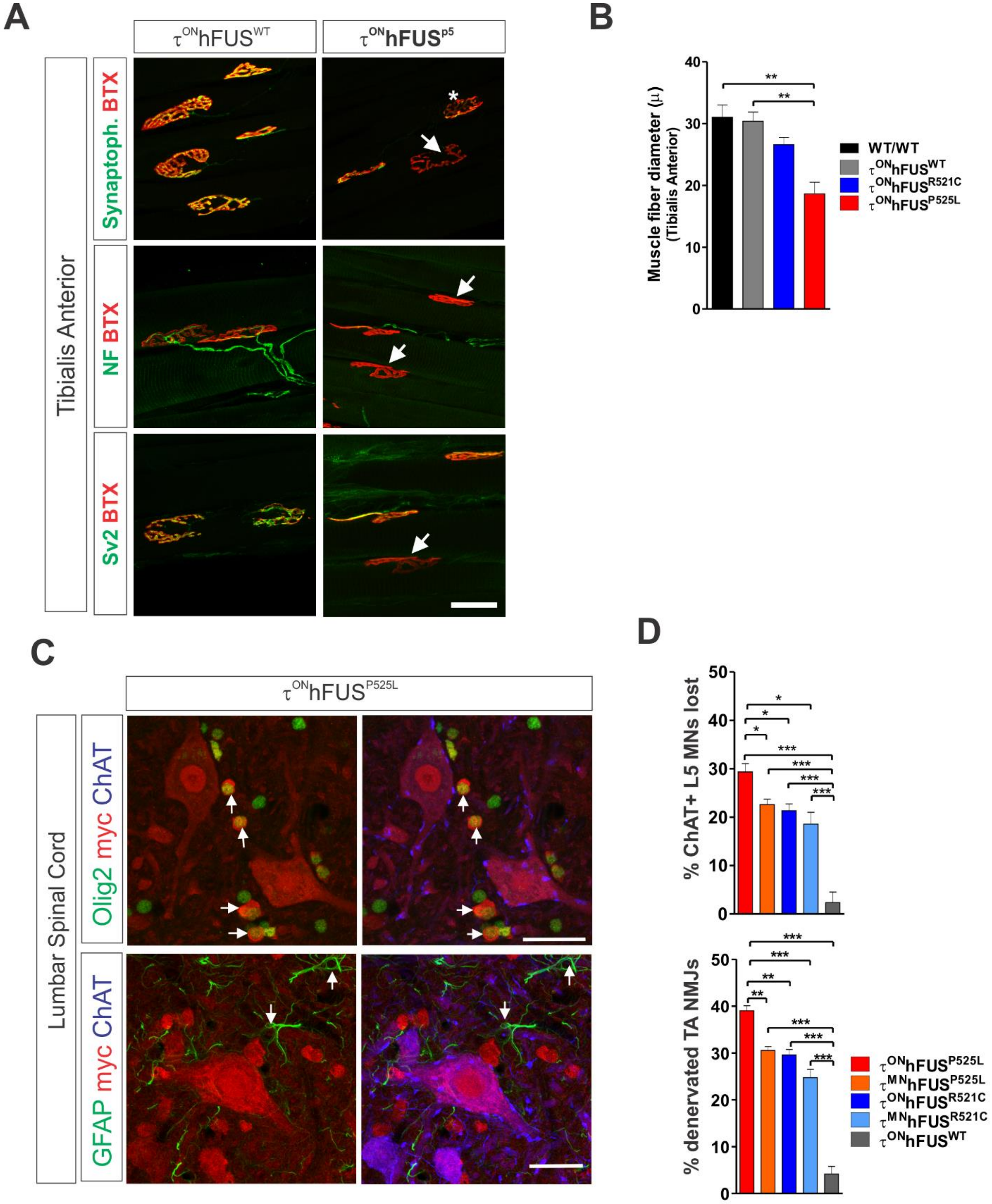
A
τ^{ON}hFUS^{WT}
τ^{ON}hFUS^{p5}
Tibialis Anterior
Synaptoph. BTX
NF BTX
Sv2 BTX
B
Muscle fiber diameter (μ)
(Tibialis Anterior)
40
30
20
10
0
**
**
WT/WT
τ^{ON}hFUS^{WT}
τ^{ON}hFUS^{R521C}
τ^{ON}hFUS^{P525L}
C
τ^{ON}hFUS^{P525L}
Lumbar Spinal Cord
Olig2 myc ChAT
GFAP myc ChAT
D
% ChAT+ L5 MNs lost
50
40
30
20
10
0
*
*
*
***
***
***
***
% denervated TA NMJs
50
40
30
20
10
0
***
***
**
**
***
***
***
τ^{ON}hFUS^{P525L}
τ^{MN}hFUS^{P525L}
τ^{ON}hFUS^{R521C}
τ^{MN}hFUS^{R521C}
τ^{ON}hFUS^{WT}

Supplementary Figure 3

(A) Immunostaining of the tibialis anterior muscle with antibodies raised against synaptophysin (synaphtoph, left), neurofilament (NF, center) and synaptic vesicle 2 (SV2, right). The white arrows indicate denervated endplates and the asterisks indicate partially denervated endplates. Scale bar = 40µm

(B) Quantification of fiber diameter in transverse sections of the tibialis anterior muscle at p360 in control mice and τ^{ON} animals. (P < 0.05, **P < 0.01, and ***P < 0.001 using one-way ANOVA with Bonferroni's post hoc test. Error bars represent SEM. N=3).

(C) Representative confocal images showing co-localization of myc and Olig2 (top) but not myc and GFAP (bottom). Scale bar = 40µm.

(D) Quantitative comparison of the percentages of MNs lost in the L5 segment (top) and the percentages of denervated NMJs (bottom) at p360 in animals expressing hFUS (either in all τ-expressing cells, τ^{ON}hFUS^{WT} , τ^{ON}hFUS^{R521C}, τ^{ON}hFUS^{P525L} or selectively in cholinergic cells, τ^{MN}hFUS^{R521C}, and τ^{MN}hFUS^{P525L}) and non-transgenic wild type controls. (P < 0.05, **P < 0.01, and ***P < 0.001 using one-way ANOVA with Bonferroni's post hoc test. Error bars represent SEM. N=3)

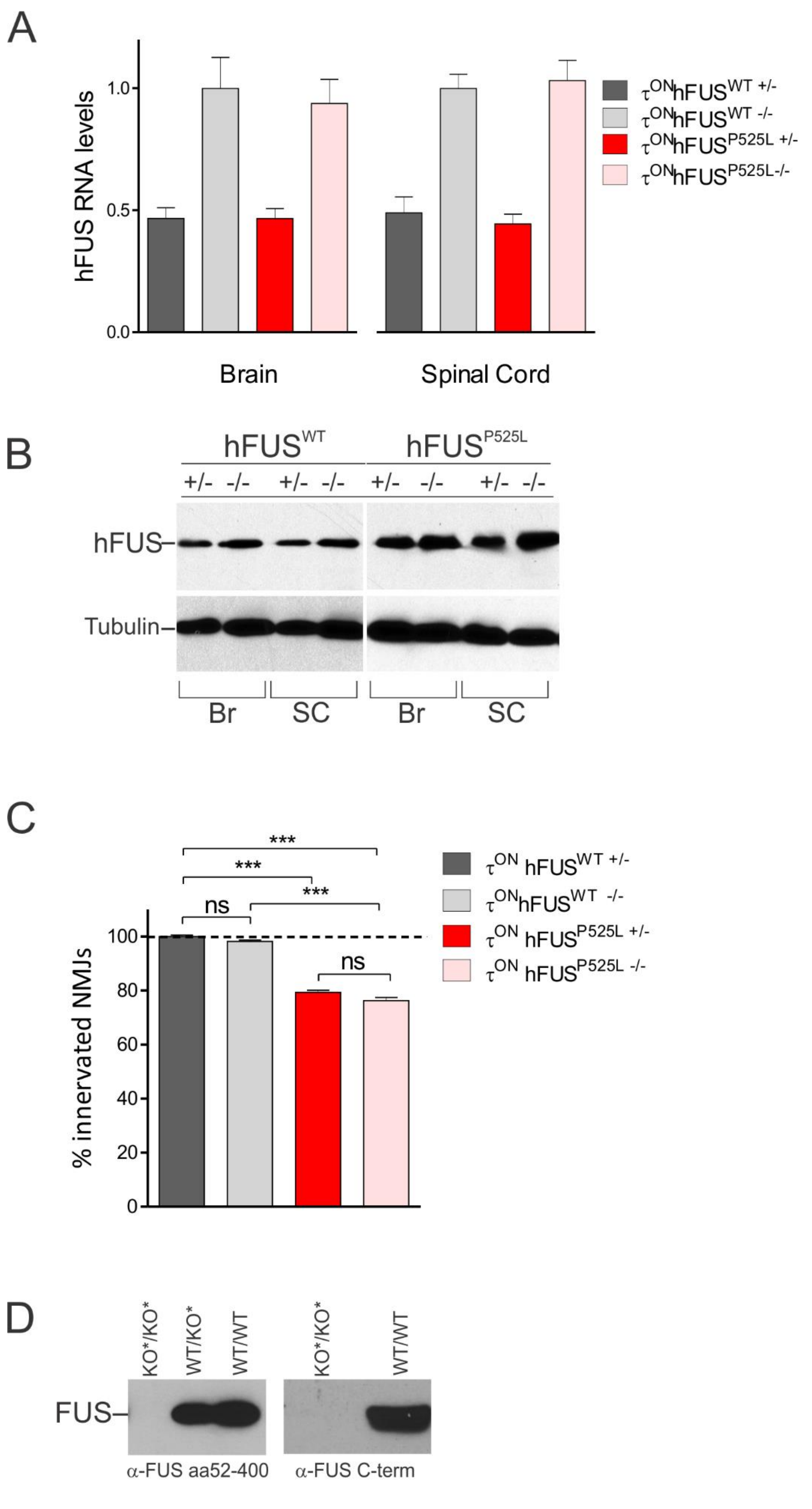
A
hFUS RNA levels
0.0
0.5
1.0
Brain
Spinal Cord
τONhFUSWT +/-
τONhFUSWT -/-
τONhFUSP525L +/-
τONhFUSP525L-/-
B
hFUSWT
hFUSP525L
+/- -/- +/- -/- +/- -/- +/- -/-
hFUS
Tubulin
Br
SC
Br
SC
C
% innervated NMJs
0
20
40
60
80
100
***
***
ns
***
ns
τON hFUSWT +/-
τONhFUSWT -/-
τON hFUSP525L +/-
τON hFUSP525L -/-
D
KO*/KO*
WT/KO*
WT/WT
KO*/KO*
WT/WT
FUS
α-FUS aa52-400
α-FUS C-term

Supplementary Figure 4

(A) RT-qPCR analysis of hFUS transcripts in total brain and total spinal cord in mice heterozygous (+/-) or homozygous (-/-) for the τ^{ON}hFUS^{WT} or τ^{ON}hFUS^{P525L} allele. (p90, N=3). Data are represented as mean and SEM.

(B) Western analysis of total spinal cord and brain extracts from mice heterozygous (+/-) or homozygous (-/-) for the τ^{ON}hFUS^{WT} or τ^{ON}hFUS^{P525L} allele.

(C) Percentage of innervated NMJs in the tibialis anterior (TA) muscle from mice heterozygous (+/-) or homozygous (-/-) for the τ^{ON}hFUS^{WT} (+/- dark gray; -/- light gray) or τ^{ON}hFUS^{P525L} (+/- red; -/- light red) allele. (All animals p90, N=3). ***P < 0.001 using one-way ANOVA with Bonferroni's post hoc test. Error bars represent SEM. N=3.

(D) Immunoblot of FUS protein in whole brains of wild type (WT/WT), heterozygous FUS null (WT/KO*), and homozygous FUS null (KO*/KO*) P0 animals. No protein products were observed in the homozygous FUS null animals when the immunoblot was probed with antibodies raised against the middle of the FUS protein (left panel, α–FUS aa 52-400, Proteintech #11570-1-AP) or the C-terminus of the FUS protein (right panel, α–FUS C-term, Santa Cruz #SC-47711).

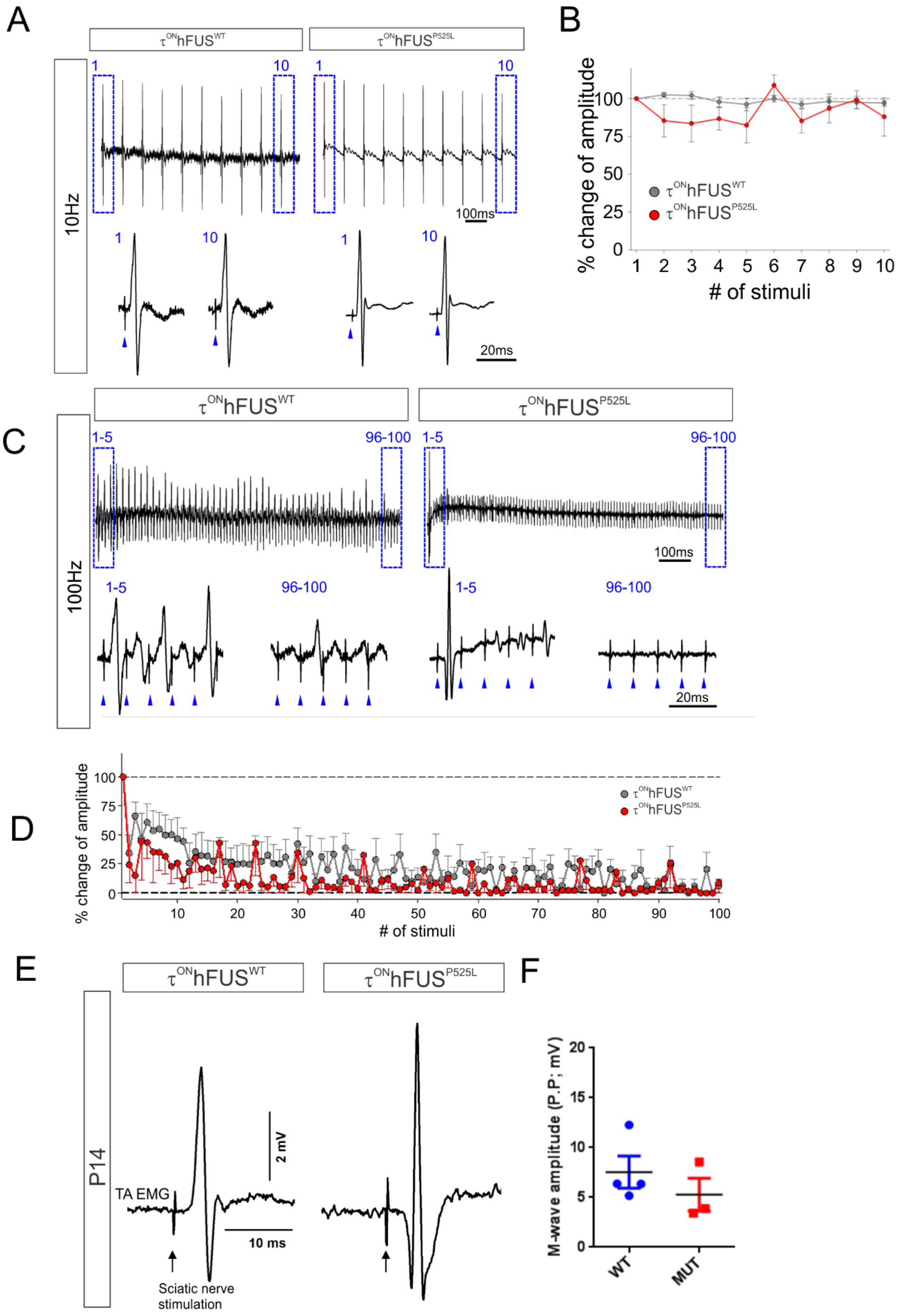

A
τONhFUSWT
τONhFUSP525L
10Hz
1
10
100ms
20ms
B
% change of amplitude
# of stimuli
C
100Hz
1-5
96-100
D
E
P14
TA EMG
Sciatic nerve stimulation
2 mV
10 ms
F
M-wave amplitude (P.P; mV)
WT
MUT

Supplementary Figure 5

(A) M-responses from a control (τ^{ON}hFUS^{WT}; upper left trace) and a mutant (τ^{ON}hFUS^{P525L}; upper right trace) mouse following 10Hz stimulation of the sciatic nerve. The first and the tenth responses are shown in an expanded time scale respectively (bottom traces).

(B) Percentage change of the M-response for all ten stimuli normalized to the first response. There was no significant change between control (gray) and mutant (red) mice.

(C) M-responses following 100Hz stimulation of the sciatic nerve (similar to those shown in A). The first and last five responses are shown in an expanded time scale below the traces in the control and mutant mice respectively. Note the complete absence of M-responses in the last five stimuli in the mutant mouse.

(D) Percentage change of the M-response as in B. Although there was no significant percentage difference between control and mutant mice, there is a marked absence of M-responses in the mutant mice indicative of NMJ neurotransmission failures.

(E) M-responses recorded from the TA of control (τ^{ON}hFUS^{WT}: left trace) and mutant (τ^{ON}hFUS^{P525L}; right trace) mice following 10Hz stimulation of the sciatic nerve at p14.

(F) M-wave amplitude recorded from the TA of a control (τ^{ON}hFUS^{WT}: blue) and a mutant (τ^{ON}hFUS^{P525L}; red) mice following 10Hz stimulation of the sciatic nerve at p14.

Supplementary Figure 6

Full Length Western Blots

Figure1

Figure1D

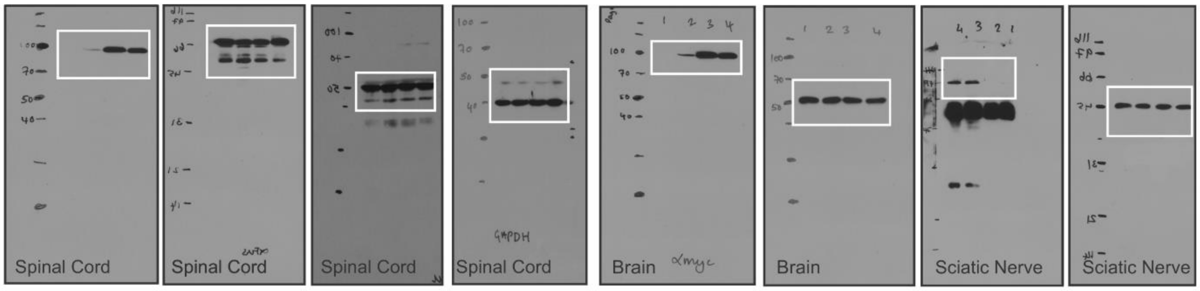

Figure 1E

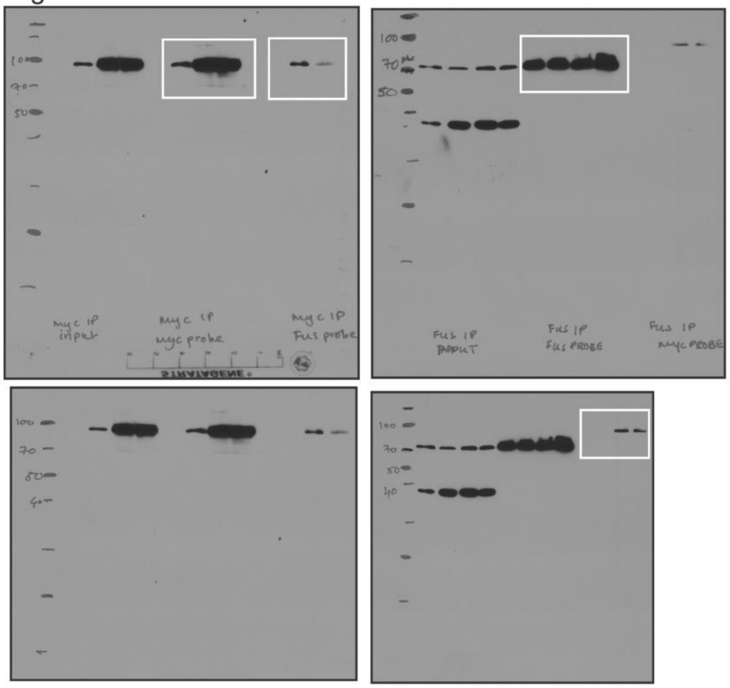

Figure1

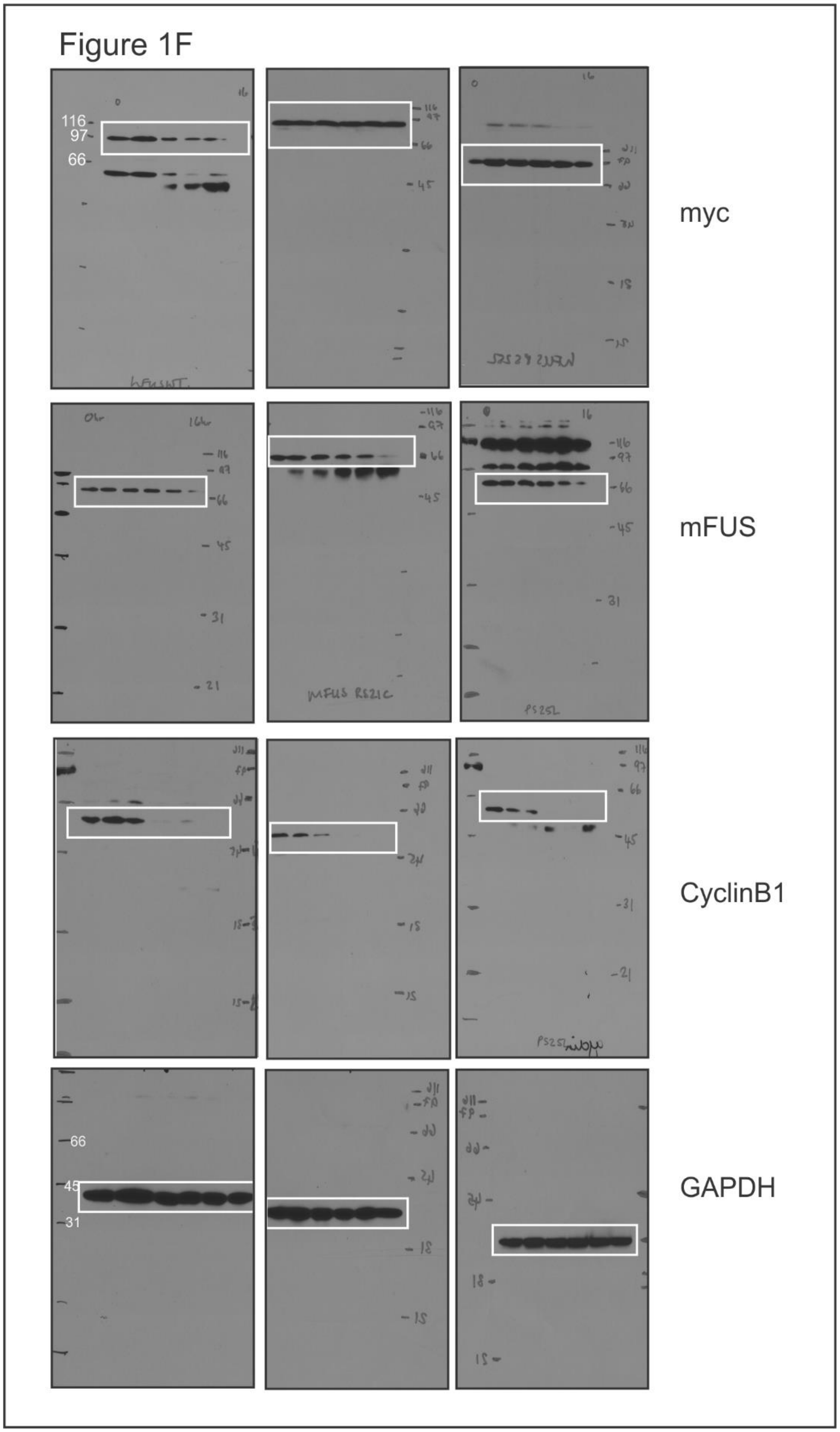

Figure 6

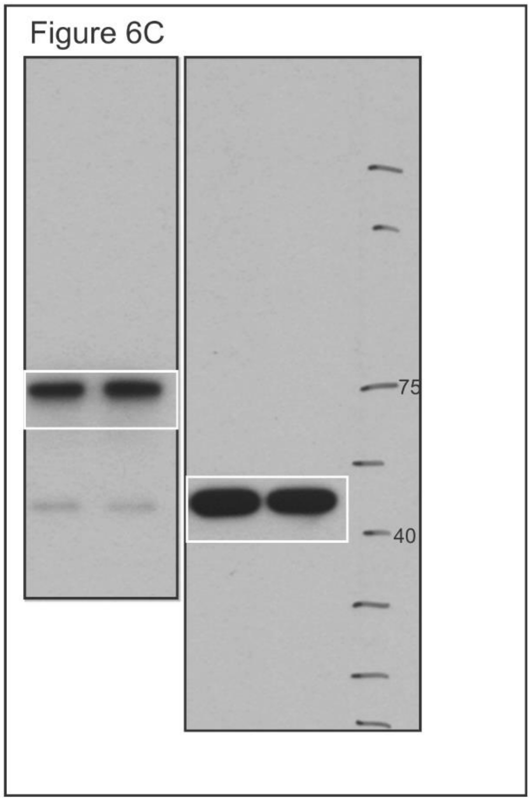

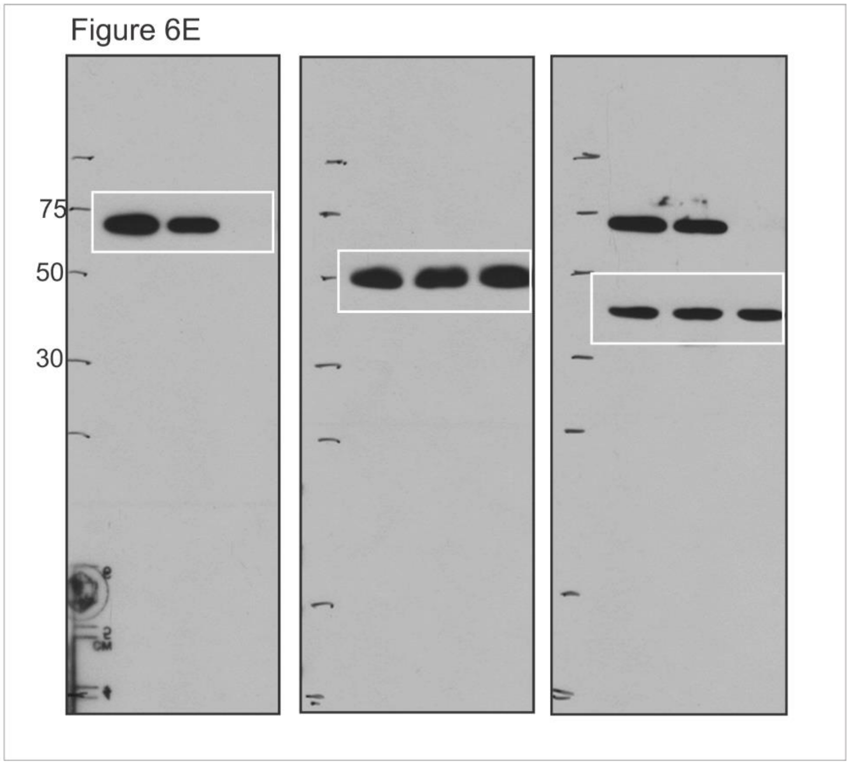

Supplementary Figure 4

S4B

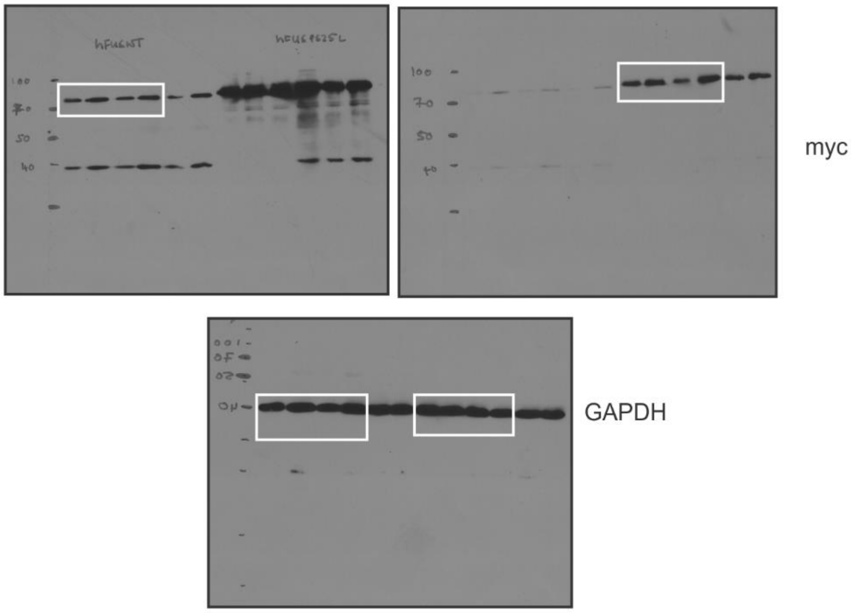

S4D

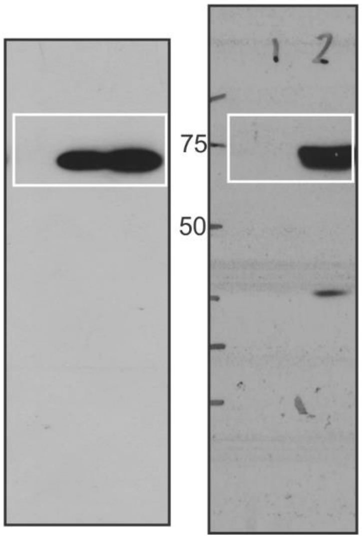

Supplementary Table 1 - Antibodies used in western blotting and immunofluorescence

Protein	Host	Source
**ChAT (1:250)**	Goat	EMD Millpore Cat AB144P
**Cyclin B1 (1:1,000)**	Rabbit	Cell Signaling 4138
**Myc-tag (1:8,000)**	Guinea Pig	Kind gift from Susan Morton/Thomas Jessell [6]
**Myc-tag (1:1,000)**	Rabbit	Cell signaling 2272
**Myc-tag (1:5,000)**	Mouse	Invitrogen R95025
**FUS (1:500 WB)**	Mouse	Santa Cruz clone 4H11 SC-47711
**FUS (1:2,000 WB)**	Rabbit	Proteintech 11570-1-AP
**FUS (1:1,000 WB, 1:200 IF)**	Rabbit	Abcam AB84078
**Parvalbumin (1:10,000)**	Chicken	Kind gift from Susan Morton/Thomas Jessell [7]
**NeuN (1:500)**	Mouse	EMD Millipore MAB377
**GAPDH (1:10,000)**	Mouse	EMD Millipore MAB374
**GFAP (1:5,000)**	Rabbit	DAKO Z0334
**Iba1 (1:5,000)**	Rabbit	WAKO 019-19741
**TDP-43 (1:10,000)**	Rabbit	Proteintech 10782-2-AP
**α-Tubulin (1:10,000)**	Mouse	Sigma-Aldrich T9026
**MBP (1:2,000)**	Rat	EMD Millipore MAB386
**Olig2 (1:10,000)**	Guinea Pig	Kind gift from Susan Morton/Thomas Jessell [8]
**VAChT (1:10,000)**	Rabbit	Kind gift from Susan Morton/Thomas Jessell (unpublished results)
**Neurofilament (1:1,000)**	Rabbit	EMD Millipore AB1987
**SV2 (1:10,000)**	Mouse	DHSB
**Synaptophysin (1:500)**	Rabbit	Invitrogen Cat 18-0130
**MMP-9 (1:1,000)**	Rabbit	Abcam AB38898
**Tau (1:10,000)**	Rabbit	DAKO A0024

Supplementary Table 2 – Primers sets used in this study

Primer	Target	Sequence	Use	Source
**$\tau^{ON/OFF}$ hFUS59 FOR**	hFUS targeted	GCCGGATGGGAAACTGAGGCTCTCC	Genotyping $\tau^{ON/OFF}$	This work
**τ^{ON} hFUS75 REV**	hFUS targeted	TGCTCCATGGTGAGGTCGCCCAAGC	Genotyping τ^{ON}	This work
**τ^{OFF} hFUS63 REV**	hFUS targeted	GGGCTGCTAAAGCGCATGCTCCAG	Genotyping τ^{OFF}	This work
**mFUS^{FLOX} 145 FOR**	mFUS (WT,FLOX)	GTCTCATAAGCCAGGGAGTACCTTCAGTGG	Genotyping FUS^{FLOX}	This work
**mFUS^{FLOX} 174 FOR**	mFUS (KO*)	TGAAGATCACCGTGAAACACTGGGCTAGG	Genotyping FUS^{FLOX}	This work
**mFUS^{FLOX} 178 REV**	mFUS(WT,FLOX,KO*)	TTTGGCTCCCAAGTTCTCACAAAAACATAATAGG	Genotyping FUS^{FLOX}	This work
**mychFUS FOR**	hFUS	AGAGTGGGAGCTACAGCCAG	qPCR	This work
**mychFUS REV**	hFUS	TTGATTGCCATAACCGCCAC	qPCR	This work
**mFUS exons1-3 FOR**	mFUS	GCTTCAAACGACTATACCCAACA	qPCR	PrimerBank
**mFUS exons1-3 REV**	mFUS	GGCCATAACCACTGTAACTCTGT	qPCR	PrimerBank
**mFUS exons12-14 FOR**	mFUS	ACTGGAAGTGTCCTAATCCTACATG	qPCR	This work
**mFUS exons12-14 REV**	mFUS	ATCTCCATAGTTTCCCCCCATA	qPCR	This work
**GAPDH FOR**	GAPDH	AATGTGTCCGTCGTGGATCTGA	qPCR	[9]
**GAPDH REV**	GAPDH	GATGCCTGCTTCACCACCTTCT	qPCR	[9]

Supplementary methods and materials

Generation of mice and mouse genetics

C57BL/6J (Stock # 000664), ChAT-Cre (Stock #018957), Protamine-Cre (Stock # 003328), Pgk1-FLPo (Stock # 011065), UBC-CreERT2 (Stock # 008085) and Hb9::GFP (Stock # 005029) mouse lines were obtained from the Jackson Laboratory (Bar Harbor, Maine, United States). ChAT-Cre and Protamine-Cre lines were back-crossed to the C57BL/6J strain for 6 generations**.** FUS^{KO} mouse line (C57BL/6 background) previously generated by a gene trap insertion in FUS exon 12[1] was a kind gift from Dr. Geoffrey Hicks.

To generate $\tau^{OFF}hFUS^{WT}$, $\tau^{OFF}hFUS^{R521C}$ and $\tau^{OFF}hFUS^{P525L}$ mouse lines the targeting constructs (described in Experimental Procedures) were electroporated into Ola/129 ES cells. Homologous recombinants were detected in approximately 35% of G418 resistant ES cell clones by Southern analysis using a probe in the 5' region, as described previously[2]. Positive ES cell clones were injected into mouse blastocysts to generate chimeras, which were then back-crossed to the C57BL/6J strain for 6 generations to establish the τ^{OFF} mouse lines. τ^{ON} mice were generated by crossing τ^{OFF} and Protamine-Cre mouse lines. τ^{MN} mice were generated by crossing τ^{OFF} and ChAT-Cre mice.

The FUS^{FLOX} mouse line was created from ES cell clone EPD0667_5_C04 (C57BL/6 background) obtained from the European Conditional Mouse Mutagenesis Program (EUCOMM). These ES cells were generated by the Helmholtz Zentrum München (Germany) as part of the International Knockout Mouse Consortium (IKMC) Project: 84575. Resultant mice carried the knockout-first version of the FUS allele (EUCOMM allele FUS^{tm1a}) and were then crossed to Pgk1-FLPo mice to produce the conditional FUS^{FLOX} line (EUCOMM allele FUS^{tm1c}) in which FUS exons 4-6 are flanked by LoxP sites. See https://www.eummcr.org/faq for details.

A novel FUS^{KO*} null allele (EUCOMM allele FUS^{tm1d}, distinct from FUS^{KO}, above) was generated by crossing FUS^{FLOX} mice to the Protamine-Cre line. Cre-mediated deletion of *FUS* exons 4-6 produces a frame shift mutation starting at codon 65 and a premature stop at codon 102 in the resulting mRNA, subjecting it to degradation by nonsense mediated decay. Any protein product, if generated, would contain only the initial 64 of 518 mouse FUS amino acids and is predicted to be non-functional.

$FUS\text{-}KO^{MN}$ mice were generated by crossing ($FUS^{FLOX/FLOX}$) to ($FUS^{KO/WT}$; $ChAT\text{-}Cre^{+/+}$) mice.

$FUS\text{-}KO^{UBC}$ mice were generated by crossing ($FUS^{FLOX/FLOX}$) to ($FUS^{KO/WT}$; $UBC\text{-}CreERT2^{+/-}$). To induce post-natal recombination of the FUS^{FLOX} allele, tamoxifen was administered to $FUS\text{-}KO^{UBC}$ and littermate control mouse pups at postnatal days 1-5 by feeding tamoxifen (dissolved in corn oil at 20mg/mL) to their nursing mother in 5 consecutive daily doses (200mg tamoxifen/kg body weight/dose) using oral gavage.

All FUS^{FLOX}, FUS^{KO*}, $FUS\text{-}KO^{MN}$ and $FUS\text{-}KO^{UBC}$ mice were maintained on a C57BL/6 background.

The genotype of these mice was determined by PCR analysis using the primers listed in **Supplemental Table 2**.

Tissue preparation for immunohistochemical analysis

Animals were deeply anesthetized using ketamine (100mg/kg, Ketaset, Pfizer) and xylazine (10mg/kg, Anased, Lloyd Laboratories) and transcardially perfused with 4% paraformaldehyde in 0.1M phosphate buffer, pH 7.4 (4% PFA).

For muscle analysis, tibialis anterior (TA), gastrocnemius (GS) and soleus (SO) muscles were dissected, post-fixed for 2 hours by immersion in 4% PFA, and washed in 1x phosphate buffered saline, pH 7.4 (PBS). Muscles were then equilibrated in a gradient of sucrose (10%-20%-30% sucrose in 0.1M phosphate buffer, pH 7.4), embedded in O.C.T. compound (Sakura, Torrance, CA) and frozen at −20°C. Consecutive sections (30µm thick) were cut using a freezing microtome (Leica CM 3050S) and stored at −80°C.

Spinal cords were isolated, immersion-fixed overnight (or 1 hour for FUS immunostaining) in 4% PFA, and washed in 1x PBS. Spinal cord segments were embedded in 4% low melting point agarose (Promega) and serial transverse sections (70µm) were cut using a vibratome (Leica VT 1000S) and processed free-floating.

Immunohistochemistry of spinal cord sections

Free-floating spinal cord sections were blocked with 5% normal donkey serum diluted in Tris buffered saline (pH 7.4) with 0.2% Triton X-100 (TBS-T) and incubated in primary antisera (**Supplemental Table 1**) diluted in TBS-T with 5% normal donkey serum overnight at room temperature. After washing with TBS-T, tissue sections were incubated for 4 hours at room temperature with species-specific secondary antibodies coupled to Alexa 488, 555, 647 (1:1,000; Life Technologies, Carlsbad, CA, USA), Cy3 or Cy5 (dilution 1:500; Jackson Immunoresearch Labs, West Grove, PA, USA). After washing with TBS-T, stained tissue sections were mounted on microscope slides in Flouromount G (Southern Biotech, Birmingham, AL, USA) and imaged using an SP5 Leica confocal microscope (Leica Microsystems, Wetzlar, Germany).

Measurement of GFAP and Iba1+ cells

A perimeter of 100µ was drawn around twenty MNs from ten 70µm lumbar spinal cord sections. The numbers of GFAP positive or Iba1 positive cells within this perimeter were then counted using ImageJ.

Electron Micrograph Analysis

Tibialis anterior neuromuscular junctions (TA-NMJs) were identified based on the presence of postsynaptic junctional folds and presynaptic nerve terminals. Micrographs were aligned using affine transformation (Fiji, NIH), and imported and segmented into TrackEM2[3]. The number of synaptic vesicles (SV), active zones, healthy mitochondria, and post-synaptic folds were quantified for both genotypes (τ^{ON}hFUS^{WT} and τ^{ON}hFUS^{P525L}). Synaptic vesicles were marked within the serial sections to prevent recounting errors. Active zones were identified as the presynaptic regions immediately opposed to postsynaptic folds. Mitochondria were considered healthy if no evidence of cristae swelling or disruption of inner and outer membranes was observed. Sarcomere length was measured between Z bands from multiple muscle fibers for each genotype.

Protein Extraction and quantitation

Whole spinal cords, brains and sciatic nerves were extracted and homogenized in SDS sample buffer (2% SDS, 10% glycerol, 5% β-mercaptoethanol, 60 mM Tris-HCl, pH 6.8, bromophenol blue). Protein extracts were quantified using the RC DC protein assay (Bio-Rad). Extracts (10-25µg) were run on a 12% SDS-PAGE gel and transferred onto a Trans-Blot transfer medium nitrocellulose membrane (Bio-Rad) using a TE77x semidry transfer unit (Hoefer). Immunoblots were probed with primary antibodies against myc, α-tubulin, FUS, TDP-43 and Tau (Supplemental Table 1). Bound antibody was detected by incubation with secondary antibodies conjugated to horseradish peroxidase (Jackson Immunoresearch) followed by chemiluminescence using a SuperSignal West Pico chemiluminescent substrate (Thermo Scientific). Signal was detected by autoradiography using Full Speed Blue sensitive medical X-ray film (Ewen Parker X-Ray Corporation). Relative protein levels were calculated using ImageJ. The full blots are shown in Supplementary Figure 6.

Immunoprecipitation

To immunoprecipitate myc-hFUS and mFUS, antibodies (Supplemental Table 1, myc and FUS) were crosslinked to protein G Dynabeads (1:1 ratio). Total spinal cords were lysed in ice-cold lysis buffer (1x PBS, 0.1% SDS, 0.5% deoxycholate, 0.5% NP-40, Complete© protease inhibitors, Roche) for 15 minutes, briefly sonicated and pre-cleared by centrifugation. Spinal cord lysates were quantified using the Pierce™ 660 Protein Assay Kit (Thermo Scientific). 200µg of total protein was mixed with 500µl of buffer A (PBS, 0.1% Triton X-100, 0.2mM PMSF), EDTA-free protease inhibitors (Roche), and 25µl of antibody-crosslinked beads. Bound proteins were eluted with 50µl of SDS sample buffer and run on a 12% SDS-PAGE gel, transferred to nitrocellulose and probed with antibodies as described above. The full blots are shown in Supplementary Figure 6.

RNA Extraction and Quantitation

Whole brains and whole spinal cords were isolated and RNA was extracted with TRIzol reagent (Invitrogen). RNA was purified using the Direct-zol RNA miniprep kit (Zymo Research) and reverse transcribed using the RevertAid first-strand cDNA synthesis kit (Fermentas). For quantitative RT-qPCR analysis, each sample was measured in triplicate with a Mastercycler ep Realplex4 (Eppendorf) PCR system and Power SYBR Green PCR Master Mix (ABI). RT-qPCR data was normalized to GAPDH. Primers used in this study are listed in Supplemental Table 2.

Protein Degradation Assay

$hFUS^{WT}$, $hFUS^{R521C}$ and $hFUS^{P25L}$ HB9::GFP transgenic mouse-derived ES cells were grown on mouse embryonic fibroblasts in ES cell medium as described previously[4,5]. Briefly, ES cell colonies were partially dissociated after 2 days and cultured in DFK5 medium. Medium was replaced at 2 days and supplemented with retinoic acid (100 nM to 2 μM) (Sigma) and Sonic hedgehog (Shh-N; 300 nM) (Curis Inc.) and EBs were cultured for 4 days.

Day 4 EBs were treated with cycloheximide (100 μg/ml) to block new protein synthesis. Medium was changed every 4h with fresh cycloheximide added. Cell lysates were collected at the indicated time points and analyzed by immunoblotting with myc, FUS, cyclin B1 and GAPDH antibodies (Supplemental Table 1). The full blots are shown in Supplementary Figure 6.

Evaluation of Motor Function

Animals ($hFUS^{WT}$, $hFUS^{P525L}$ and wild type littermate controls) were placed on a wire cage top, which was then inverted and suspended above the home cage. The latency to when the animal falls was recorded. The test was performed three days per week with five trials per session over a period of 3 weeks.

Supplementary References

1 Hicks, G. G. *et al.* Fus deficiency in mice results in defective B-lymphocyte development and activation, high levels of chromosomal instability and perinatal death. *Nat Genet* **24**, 175-179 (2000).

2 Bibel, M. & Barde, Y. A. Neurotrophins: key regulators of cell fate and cell shape in the vertebrate nervous system. *Genes Dev* **14**, 2919-2937 (2000).

3 Cardona, A. *et al.* TrakEM2 software for neural circuit reconstruction. *PLoS One* **7**, (2012).

4 Wichterle, H., Lieberam, I., Porter, J. A. & Jessell, T. M. Directed differentiation of embryonic stem cells into motor neurons. *Cell* **110**, 385-397 (2002).

5 Wichterle, H. & Peljto, M. Differentiation of mouse embryonic stem cells to spinal motor neurons. *Current protocols in stem cell biology* **Chapter 1**, Unit 1H.1.1-1H.1.9, (2008).

6 Arber, S. *et al.* Requirement for the homeobox gene Hb9 in the consolidation of motor neuron identity. *Neuron* **23**, 659-674 (1999).

7 de Nooij, J. C., Doobar, S. & Jessell, T. M. Etv1 inactivation reveals proprioceptor subclasses that reflect the level of NT3 expression in muscle targets. *Neuron* **77**, 1055-1068, (2013).

8 Novitch, B. G., Wichterle, H., Jessell, T. M. & Sockanathan, S. A requirement for retinoic acid-mediated transcriptional activation in ventral neural patterning and motor neuron specification. *Neuron* **40**, 81-95 (2003).

9 Ruggiu, M. *et al.* A role for SMN exon 7 splicing in the selective vulnerability of motor neurons in spinal muscular atrophy. *Mol Cell Biol* **32**, 126-138, (2012).
